# Bioprocessing of common pulses changed seed microstructures, and improved dipeptidyl peptidase-IV and α-glucosidase inhibitory activities

**DOI:** 10.1038/s41598-019-51547-5

**Published:** 2019-10-25

**Authors:** Elisa Di Stefano, Apollinaire Tsopmo, Teresa Oliviero, Vincenzo Fogliano, Chibuike C. Udenigwe

**Affiliations:** 1 School of Nutrition Sciences, University of Ottawa, Ottawa, Ontario K1H 8M5 Canada; 2 Food Quality and Design Group, Wageningen University and Research, P.O. Box 8129, 6700 EV Wageningen, The Netherlands; 3 Food Science and Nutrition Program, Department of Chemistry, Carleton University, Ottawa, Ontario K1S 5B6 Canada; 4 Department of Chemistry and Biomolecular Sciences, University of Ottawa, Ottawa, Ontario K1N 5E3 Canada

**Keywords:** Biochemistry, Physiology

## Abstract

Type 2 diabetes mellitus (T2DM) is a leading cause of death globally. T2DM patients experience glucose intolerance, and inhibitors of dipeptidyl peptidase IV (DPP-IV) and α-glucosidase are used as drugs for T2DM management. DPP-IV and α-glucosidase inhibitors are also naturally contained in foods, but their potency can be affected by the food matrix and processing methods. In this study, germination and solid-state fermentation (SSF) were used to alter pulse seed microstructures, to convert compounds into more bioactive forms, and to improve their bioaccessibility. Germination substantially modified the seed microstructure, protein digestibility, contents and profiles of phenolic compounds in all the pulses. It also increased DPP-IV and α-glucosidase inhibitory activities in chickpeas, faba beans and yellow peas. Compared to germination, SSF with *Lactobacillus plantarum* changed the content and the profile of phenolic compounds mainly in yellow peas and green lentils because of greater disruption of the seed cell wall. In the same pulses, heat treatment and SSF of flour increased DPP-IV and α-glucosidase inhibitory activities. The results of this study suggest that germination and SSF with *L. plantarum* are effective and simple methods for modulating phenolic and protein profiles of common pulses and improve the action on DPP-IV and α-glucosidase.

## Introduction

Diabetes is currently one of the ten leading causes of death globally and its morbidity is expected to reach 10.4% of the world population by 2040^1,2^. Type 2 diabetes mellitus (T2DM) is the most common condition, representing about 90% of diagnosed diabetes in high-income countries, and its development is highly influenced by environmental and dietary factors^3^. T2DM patients suffer from many metabolic anomalies such as insulin resistance and deficient pancreatic β-cells insulin secretion, which in turn cause difficulty in maintaining blood glucose homeostasis and glucose intolerance^4,5^. Decreasing postprandial glucose absorption by inhibiting key digestive enzymes involved in glucose regulation, or by facilitating the action of gut hormones involved in insulin secretion has therefore become a target for many anti-diabetic drugs. The human digestive enzymes, α-glucosidase, α-amylase and dipeptidyl peptidase IV (DPP-IV), play a central role in postprandial glucose regulation. α-Glucosidase (α-D-glucoside glucohydrolase, EC 3.2.1.20) is the primary enzyme involved in the hydrolysis of terminal α-1,4-linked glucose moieties from polysaccharides in the human body^6^. DPP-IV is a ubiquitous enzyme that selectively cleave glucagon-like peptide-1 (GLP-1) and glucose-dependent insulinotropic peptide (GIP). GLP-1 and GIP are known as incretin hormones; they trigger the secretion by 60% of the insulin released during meal consumption, the so-called “incretin response”^7^. DPP-IV drastically impair the metabolism of incretin hormones by converting about 85% of the biologically active hormones to their inactive forms in the human body^8,9^. Drugs or food-derived bioactive compounds able to inhibit α-glucosidase and DPP-IV are therefore used as obvious strategy in T2DM management^10,11^.

Pulses are an excellent source of dietary proteins and other nutrients, and play a major role in the diets of many developing countries. They are regarded as low glycaemic index food, and recent *in silico* and *in vitro* studies have shown DPP-IV and α-glucosidase inhibitory properties of peptides and phenolic extracts derived from pulses^12–19^. Moreover, pulses contain other components with beneficial effects on glycaemic regulation, such as complex carbohydrates and resistant starches^20^. Processing of plant material can improve bioavailability of nutrients and bioactive compounds by disrupting the plant cell wall, dissociating nutrient-matrix complexes, or biotransforming them into more active forms. Improved bioaccessibility was indeed observed in processed protein and phenolic extracts from pulses^13–15,21–23^, but the bioaccessibility of these components when concurrently present in a food matrix is still not clear. Thermal treatment can either be beneficial or detrimental for nutrient bioavailability, with particular impact on proteins. Heat can inactivate protease inhibitors and denature proteins in seeds, potentially making them more available to digestive enzymes, or also encourage aggregation and prevent enzyme accessibility when starch gelatinization occurs^14,24,25^. Desiccated commercial legumes are in a metabolically dormant state, with seed coats rich in protective compounds (e.g. phenolic compounds, lignin, cutin) which are not digestible for human digestive enzymes. Intactness and porosity of the cell wall therefore play a major role in the bioavailability of micro- and macronutrients of plant seeds^25–27^. Soaking legume seeds (water imbibition) ends their dormancy stage and initiates the germination process, with significant changes in protein profile and dramatic impact on nutrients composition^28^. Germination also triggers the synthesis of phenolic compounds, which are needed for their antioxidant activity in the early stages of germination, and for the structural growth of the plant in later stages^29,30^. Furthermore, germination of legumes has been used for generating bioactive peptides with DPP-IV and α-glucosidase inhibitory activity^13,31^. Fermentation has also been successfully used as a sustainable method for improving protein digestibility, releasing bioactive peptides from the native protein sequence, degrading tannins, increasing soluble phenolic compounds and, thus, improving the bioactivity of plant materials^22,32–34^.

The objective of this study was to investigate the effect of physical and biological processing on the bioaccessibility of nutrients and bioactive molecules with α-glucosidase and DPP-IV inhibitory activities in five common pulses. Soaking, heating, grinding, SSF with *Lactobacillus plantarum*, and germination were used as bioprocessing methods, alone or combined, for treating chickpeas, kidney beans, faba beans, green lentils, and yellow peas. Detailed study on the microstructure, protein and phenolic profiles of bioprocessed yellow peas and green lentils was conducted to provide further evidence on how the bioprocesses modulate the content and chemical form of specific bioactive molecules. The ultimate goal was to select an optimal bioprocessing method (i.e. the one leading to the highest α-glucosidase and DPP-IV inhibition *in vitro*) to design functional ingredients for T2DM prevention.

## Results

### Efficiency of bioprocessing

The pH significantly decreased for all samples during SSF, with ground samples showing the most prominent decrease in all the pulses. The pH value dropped from 5.9 ± 0.2 to 4.3 ± 0.6 in soaked and cracked pulse seed samples, from 6.3 ± 0.1 to 4.5 ± 0.2 in autoclaved seed samples, and from 6.1 ± 0.1 to 3.9 ± 0.1 in ground samples. The bacterial population of *L. plantarum* increased to 9.2 log colony forming units (CFU)/mL in soaked samples, to 9.3 log CFU/mL in autoclaved samples and decreased to 6.1 CFU/mL in ground samples. Germination efficiency, calculated as percentage of germinated seeds over the total number of seeds was maximum (100%) for green lentils, both at day 3 and 5 of germination, followed by yellow peas (97.7 ± 0.5% and 99.3 ± 1.0% at days 3 and 5, respectively) and faba beans (90.0 ± 1.7% and 91.9 ± 7.6% at days 3 and 5, respectively). Kidney bean and chickpea germination efficiency had higher variability (77.6 ± 12.9% and 90.1 ± 2.4% at days 3 and 5, respectively for kidney beans and 63.5 ± 1.3% and 65.1 ± 5.2% for chickpeas), which makes these two germinated pulses less befitting for further application in our study.

### Effect of bioprocessing on bioaccessibility of bioactive compounds and inhibition of glucose-regulating enzymes

#### Total phenolic content and degree of hydrolysis of proteins

Total phenolic content and protein degree of hydrolysis values for physically and biologically processed pulses, before and after simulated gastrointestinal digestion, are shown in Table 1. Phenolic contents before bioprocessing (germination and solid-state fermentation) differed between pulses, with the highest values observed for faba beans, kidney beans and green lentils. Grinding the pulses to flour prior to bioprocessing led to an increase in total phenolic content (TPC) in faba beans, kidney beans and green lentils. During germination, a gradual and significant increase in TPC before *in vitro* digestion was observed in faba beans and yellow peas, while kidney beans showed the opposite trend. The highest increase was observed for faba beans, with TPC values almost doubling during 5 days of germination. Simulated gastrointestinal digestion led to a significant increase in TPC in all the germinated pulses, but no significant difference in TPC was observed between various germination times of each digested pulse. Germination also induced a significant increase in protein digestibility, measured as degree of hydrolysis (DH). The highest increase prior to *in vitro* digestion was observed in yellow peas, faba beans and green lentils, with digestibility increasing by two or three folds. Simulated gastrointestinal digestion also led to a substantial increase in DH in all the pulses, when compared to the undigested samples. However, the changes in DH caused by germination were not significant after gastrointestinal digestion, with the exception of yellow peas. In fact, digested germinated yellow peas showed the overall highest increase in protein digestibility among all samples.

**Table 1:** Total phenolic content (TPC), expressed as gallic acid equivalent/g DW, and protein degree of hydrolysis (DH), expressed as milliequivalent Ser-NH_2_/g DW, of bioprocessed pulses (mean ± SD, n = 3).

		GERMINATION						SOLID STATE FERMENTATION											
Pulse		Day 0		Day 3		Day 5		S		S + SSF		HT		HT + SSF		G		G + SSF	
		TPC	DH	TPC	DH	TPC	DH	TPC	DH	TPC	DH	TPC	DH	TPC	DH	TPC	DH	TPC	DH
Chickpeas	ND	1.72 ± 0.15	20.49 ± 3.01^1^	2.41 ± 0.17	24.11 ± 3.15^2^	2.16 ± 0.19	32.01 ± 5.64^12^	1.10 ± 0.47^a^	16.52 ± 1.26^123^	2.21 ± 0.06	17.04 ± 0.18^45^	1.79 ± 0.45^b^	12.08 ± 0.35^1467^	1.79 ± 0.09^c^	10.57 ± 0.65^2589^	1.86 ± 1.04	17.13 ± 0.08^68^	3.04 ± 0.10^abc^	21.18 ± 1.00^379^
	SGID	5.39 ± 0.23	103.75 ± 2.31	5.43 ± 0.20	111.35 ±10.26	4.36 ± 0.52	109.60 ± 4.94	3.19 ± 0.80	97.37 ± 8.59	2.44 ± 0.26	87.62 ± 7.28	2.61 ± 0.25	100.92 ± 2.04	3.13 ± 0.55	102.00 ± 17.33	3.18 ± 0.24	94.13 ± 5.01	3.59 ± 0.29	92.36 ± 1.19
Faba Bean	ND	2.40 ± 0.24 ^a^	23.93 ± 1.38^1^	3.22 ± 0.20 ^a^	27.49 ± 2.35^2^	4.33 ± 0.43 ^a^	47.44 ± 17.71^12^	2.78 ± 0.96^a^	17.42 ± 4.61^123^	3.40 ± 0.91	17.98 ± 1.67^456^	3.75 ± 0.34^bc^	12.66 ± 1.30^1478^	2.59 ± 0.24^bd^	11.07 ± 0.77^259*^	4.05 ± 0.69^a^	19.14 ± 1.82^79^	3.87 ± 0.58^cd^	22.41 ± 1.75^368*^
	SGID	5.71 ± 0.62	137.84 ± 5.11	7.12 ± 0.41	134.42 ± 1.65	7.26 ± 1.84	138.23 ± 4.48	4.80 ± 0.44	119.98 ± 6.80	4.33 ± 0.38	113.74 ±10.38^1^	5.51 ± 1.99	113.68 ± 6.80	5.58 ± 2.08	104.00 ± 2.93^2^	6.63 ± 0.45	113.10 ± 0.24	5.17 ± 1.94	143.84 ± 27.63^12^
Kidney Beans	ND	3.24 ± 0.42 ^a^	18.94 ± 1.08^12^	2.65 ± 0.92	24.73 ± 1.14^1^	2.52 ± 0.13 ^a^	27.87 ± 1.72^2^	2.14 ± 0.81^abc^	17.78 ± 3.91^12^	3.74 ± 0.15^ade^	16.24 ± 2.15^34^	2.40 ± 0.25^df^	10.34 ± 0.89^1356^	2.15 ± 0.48^egh^	9.53 ± 0.44^2478^	3.35 ± 0.30^bg^	17.16 ± 0.13^57^	4.12 ± 0.35^cfh^	19.10 ± 2.55^68^
	SGID	3.27 ± 0.51	79.11 ± 9.09	3.07 ± 1.00	73.27 ± 5.35	3.61 ± 0.52	71.06 ± 7.33	3.27 ± 0.44	79.41 ± 14.97	3.54 ± 0.65	98.11 ± 4.57^1^	3.14 ± 0.38	95.17 ±17.55^2^	2.93 ± 0.09	85.17 ± 3.16^3^	2.76 ± 0.54	83.31 ±13.50^4^	2.87 ± 0.39	51.12±33.59^1234^
Green Lentils	ND	2.41 ± 0.08	23.80 ± 1.55^1^	2.62 ± 0.10	25.91 ± 0.94^2^	2.77 ± 0.20	43.11 ± 2.21^12^	2.11 ± 0.40^abc^	23.57 ± 3.99^12^	2.51 ± 0.39^de^	22.33 ± 0.70^34^	3.99 ± 0.47^adf^	16.89 ± 0.81^1356^	2.90 ± 0.19^g^	16.29 ± 1.28^2478^	3.45 ± 0.42^bc^	24.48 ± 2.09^57^	6.06 ± 0.88^cefg^	23.03 ± 0.63^68^
	SGID	5.46 ± 0.25	117.41 ± 11.99	5.71 ± 0.37	116.02 ± 6.23	4.61 ± 0.82	120.42 ± 5.70	4.85 ± 0.43	118.21 ± 19.82	4.02 ± 0.78	121.96 ± 5.48	4.07 ± 1.26	126.91 ± 10.47	3.39 ± 1.28	122.01 ± 6.01	4.12 ± 0.75	114.99 ± 23.92	3.75 ± 0.18	111.31 ± 5.66
Yellow Peas	ND	1.41 ± 0.06 ^a^	17.33 ± 0.79^1^	1.94 ± 0.27	36.15 ± 1.05^1^	2.31 ± 0.11 ^a^	42.20 ± 1.18^1^	1.98 ± 0.13	16.78 ± 1.61^123^	2.56 ± 0.23^a^	17.81 ± 0.76^456^	1.60 ± 0.26	9.91 ± 0.75^147^	1.23 ± 0.32^ab^	9.05 ± 0.25^256^	1.91 ± 0.22	14.00 ± 0.70^8^	2.68 ± 1.00^b^	24.65 ± 0.78^3678^
	SGID	4.78 ± 0.44	98.23 ± 6.72^1^	5.38 ± 0.43	102.27 ± 3.23^2^	5.11 ± 0.52	125.54 ± 8.77^12^	3.39 ± 0.76	90.50 ±16.04	3.10 ± 0.47	117.24 ±10.49	2.99 ± 0.52	95.35 ± 5.28	3.45 ± 1.33	87.90 ± 5.10	3.35 ± 0.16	90.82 ± 4.79	4.49 ± 0.57	115.56 ± 7.02

Germination was performed for 0 (Day 0), 3 (Day 3) and 5 (Day 5) days in the darkness at 23 °C. Solid state fermentation (SSF) with L. plantarum ATCC 8014 for 48 hours at 37 °C. Prior SSF, samples were either soaked (S) overnight, heat treated 121 °C for 15 min (HT), or grinded into flour (G). ND: non digested; SGID: simulated gastrointestinal digestion. For each row and bioprocessing (germination and fermentation), TPC values marked with the same letter are significantly different (p < 0.05); DH values marked with ^n^ are significantly different (p < 0.05).

The pre-treatment applied before SSF substantially influenced the ability of *L. plantarum* to ferment the substrate (i.e., the pre-treated sample). Soaking and grinding increased TPC and DH with SSF in chickpea and yellow pea, while a decrease was observed after SSF of heat-treated samples. A significant increase in TPC during 48 h fermentation with *L. plantarum* was observed for ground green lentil samples and soaked kidney beans, while SSF of heat-treated samples led to a significant decrease in TPC in faba beans. Protein digestibility was higher in soaked and ground unfermented pulses compared to the heat-treated ones. SSF of ground samples led to an increase in DH in all the pulses except for green lentils, with significant values observed for yellow peas. *In vitro* digestion induced a large increase in all TPC and DH values, with the exception of kidney beans, masking the effect of the bioprocessing. Despite the bioprocessing applied, faba beans, kidney beans and green lentils had the highest TPC, while green lentils and yellow peas had the highest values for protein digestibility.

#### DPP-IV inhibitory activity

**Figure 1:**
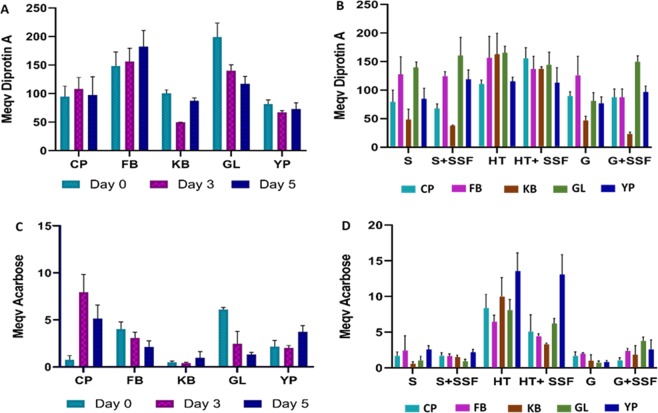
Enzyme inhibitory activity of bioprocessed pulses after *in vitro* digestion. (Panels A,B): DPP-IV inhibition of germinated (**A**) and solid state fermented (**B**) pulse samples. Values are expressed as milliequivalent Diprotin A (mM Diprotin A Eqv/100 mg DW). (Panel C,D): α-glucosidase inhibition of germinated (**C**) and solid state fermented (**D**) pulse samples. Values are expressed as milliequivalent Acarbose (mM Acarbose Evq/100 mg DW). CP: chickpea, FB: faba bean, KB: kidney bean, GL: green lentil, YP: yellow pea; S: soaked, SSF: solid state fermentation, HT: heat treatment, G: grinding. Data are expressed as mean ± SD of three independent replicates.

Results on the DPP-IV inhibition assay are reported in Supplementary Table S1. Results converted into diprotin A equivalents are shown in Fig. 1, Panels A and B. The DPP-IV inhibition did not deviate significantly from the average value of 57% inhibition. Regardless the processing method, green lentils and faba beans showed higher DPP-IV inhibitory activity compared to the other pulses. Germination had a negative impact on the bioactivity, with the exception of faba beans. Among the processing methods, the heat treatment appeared to be the most impactful in enhancing the bioactivities. The combination of heat treatment and SSF with *L. plantarum* appeared to be beneficial only for chickpeas, while a slight decrease in bioactivity was observed for the other pulses. Soaking and grinding of samples had mild effect on DPP-IV inhibition, while their combination with SSF appeared to be beneficial in the case of green lentils and yellow peas. Overall, yellow peas and green lentils were the most susceptible pulses to the activity of *L. plantarum*, with beneficial outcome on their bioactivity. SSF of ground pulses induced an increase in DPP-IV inhibitory activity for green lentils and yellow peas. On the other hand, the combination of grinding and SSF led to a significant decrease in bioactivity for kidney beans.

#### α-glucosidase inhibitory activity

Results of the α-glucosidase inhibition assay are reported in Supplementary Tables S2. Results converted in Acarbose equivalents are shown in Fig. 1, Panels C and D. As observed for DPP-IV, unprocessed green lentil showed the highest inhibitory activity against α-glucosidase. α-Glucosidase inhibitory activity varied substantially between pulses after germination (Fig. 1, Panel C). In chickpeas, germination for 3 days caused a significant increase of about 36% of the bioactivity, which then slightly decreased at day 5 of further germination. Likewise, germination of kidney beans and yellow peas led to a moderate increase in α-glucosidase inhibitions. Opposite trend was observed for faba beans and green lentils, with a significant decrease of about 24% of the bioactivity after 5 days of germination in the latter case. The pre-treatment applied before SSF had a substantial influence on α-glucosidase inhibition (Fig. 1, Panel D). SSF of heat-treated samples led to a decrease in bioactivity in all the pulses, while fermentation of ground flour greatly increased the bioactivity, except for chickpeas. The most remarkable impact of SSF was observed in ground green lentil and yellow pea samples, where the bioprocessing led to an increase of 18% and 17% of α-glucosidase inhibitory activity, respectively. Heat treatment caused an increase in bioactivity in all the pulses, when compared to the other treatments.

### Changes in phenolic and protein profiles, and microstructures of yellow peas and green lentils

#### Protein profile (SDS-PAGE)

**Figure 2:**
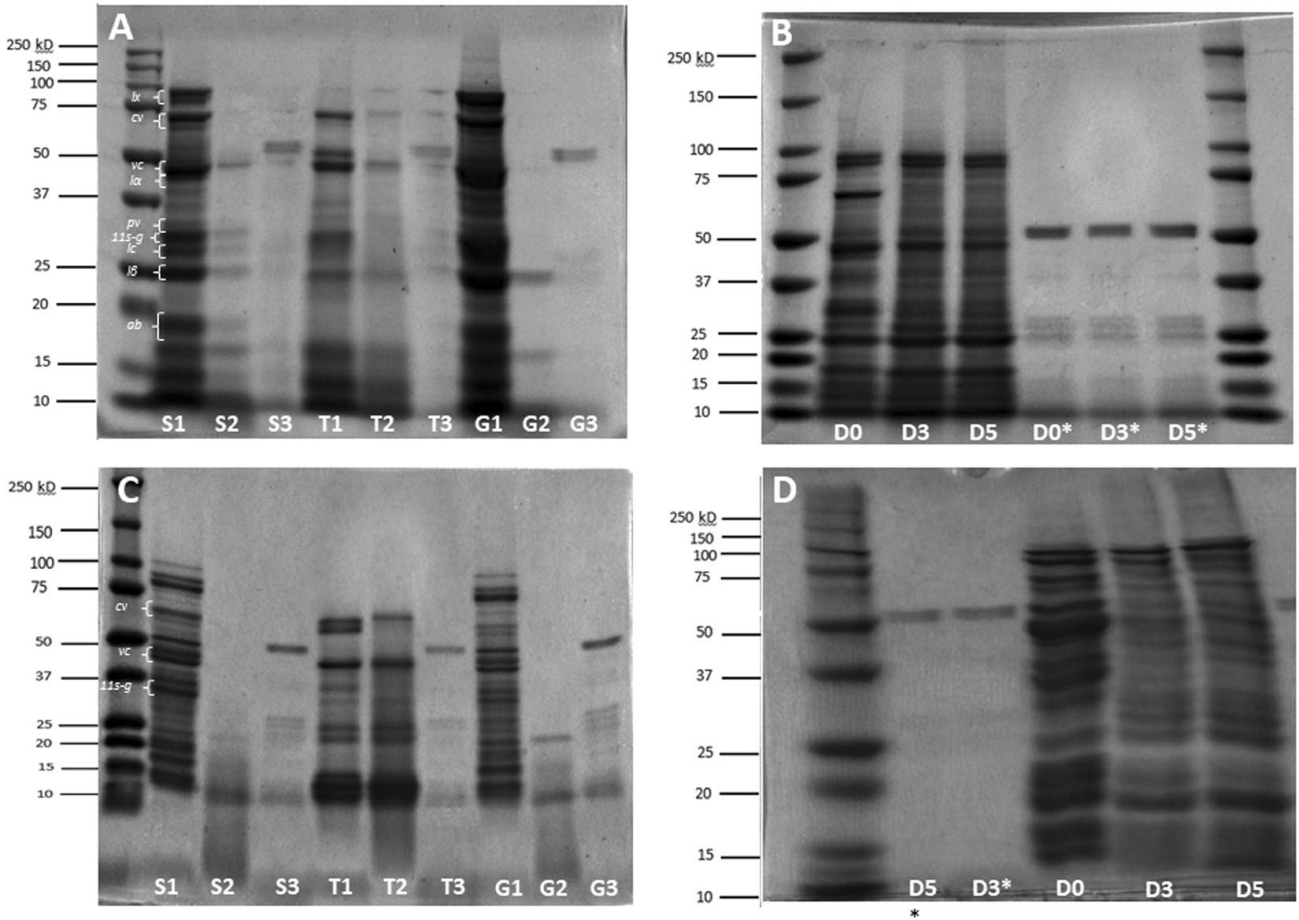
SDS-PAGE profiles of water-soluble extracts obtained from yellow peas (Panels A,B) and green lentils (Panels C,D) by solid state fermentation with Lactobacillus plantarum at 37 °C for 48 h (Panels A,C) and germination (Panel B,D). (Panels A,C): S1–S3: soaking (S1) followed by SSF (S2) and simulated gastrointestinal digestion (S3); A1–A3: autoclaving (A1) followed by SSF (A2) and simulated gastrointestinal digestion (A3); G1-–G3: grinding (G1) followed by SSF (G2) and simulated gastrointestinal digestion (G3). Panel B,D: D0-D3: day 0 (D0), 3 (D3) and 5 (D5) of germination at room temperature (23 °C) in the darkness. *ab*: albumin fraction; *lβ*: legumin β; *lc*: lectin; *lα*: legumin α; *pv*: provicilin; *vc*: vicilin; *cv*: convicilin; *lx*: lipoxygenase; *11s-g*: 11s globulin.

The protein profile of fermented and germinated yellow pea extracts are shown in Fig. 2. Panels A and C show protein profiles of the seed samples upon soaking, heat treatment and grinding, alone or in combination with SSF (*L. plantarum*). When considering the unfermented samples (S1, T1, G1), more bands can be observed in the ground seeds (G1), while heat treatment (T1) seemed to have substantially decreased the number and intensity of the protein bands. Yellow pea proteins were characterized by a variety of polypeptide subunits in the molecular weight range of 10 to 97 kDa, with major subunits at 17–19 kDa (albumin fraction), 25 kDa (legumin β), ~30 kDa (lectin), 40 kDa (legumin α), 35 and 50 kDa (vicilin), 70 kDa (convicilin), and 97 kDa (lipoxygenase), which were identified based on literature^35–38^. Albumin, lipoxygenase and legumin alpha were degraded substantially in ground-SSF yellow pea samples (G2), showing a 10- to 26-fold decrease in band intensities. Soaked-SSF pea samples (S2) also showed a 3- to 9-fold decrease in band intensities for the same proteins. SSF appeared to be less effective on heat treated samples (T2) when compared to the other pre-treatments, and this was especially evident in green lentils (Fig. 2C), where no decrease greater than 1-fold was observed for the major proteins. In accordance with the results on protein degree of hydrolysis (Table 1), a combination of grinding and SSF led to the most marked decrease in protein bands, possibly due to extensive proteolytic activity during fermentation. Simulated gastrointestinal digestion heavily affected the intensity of the bands, especially in green lentils, suggesting an intense proteolytic activity of digestive enzymes on pulse proteins. Besides an overall decrease in bands intensity over time, it is interesting to notice the changes in certain groups of proteins, and not others, during germination. In pea proteins (Fig. 2, Panel B), the major bands of convicilin, vicilin and provicilin (32 kDa) showed a 1.5-fold decrease in band intensity after 3 days of germination. Similar to the pea proteins, the bands appeared very intense in soaked (S1) and ground (G1) samples, and significantly less intense after heat treatment (T1). Similar to the pea proteins, it is possible that the intense heat treatment caused denaturation and aggregation of proteins, with subsequent decrease in solubility. Solid-state fermentation appeared to be very effective in degrading proteins, since only a few bands with weak intensities were present in profiles of S2 and G2 samples. On the other hand, SSF of heat-treated lentils did not significantly affect the protein profile, except for the ~50 kDa, which showed a 2-fold decrease in T2. Germination of green lentils resulted in an overall decrease in band intensities (Fig. 2, Panel D). As expected, simulated gastrointestinal digestion had an intense effect on all samples, with very little protein bands present after the treatment (Fig. 2: S3, T3, G3, DO*, D3*, D5*).

#### Identification and quantification of phenolic compounds in bioprocessed green lentil and yellow pea

A total of 12 and 11 phenolic compounds were identified in green lentil and yellow pea, respectively (Supplementary Table S3). When looking at the green lentil phenolic profile (Supplementary Fig. S1), compounds with the highest intensities corresponded to flavonoids, with predominance of kaempferol derivatives. In lentils, both germination and fermentation led to a significant decrease in intensities of kaempferol tetraglycoside and kaempferol triglycoside, which corresponded to an increase in intensities of three flavonoid compounds in the germinated samples and that of the kaempferol glycoside in fermented samples. In yellow peas, the highest peaks were identified to be vanillic acid and flavonoids (luteolin glucoside, kaempferol derivative, and flavonoid derivative). Germination for 5 days caused a substantial increase in protocatechuic acid, vanillic acid, chlorogenic acid and flavonoid derivatives. Two peaks were detected at the beginning of the yellow pea germination (day 0) at approximately R_t_ of 15 and 18 min, which significantly decreased as germination progressed, while four new peaks appeared at R_t_ of 14, 15.4, 16.7 and 19 min at day 5 (Supplementary Fig. S1). Peaks corresponding to chlorogenic acid, luteolin glucoside luteolin-6-C-glucoside and flavonoid derivative were not retained after simulated gastrointestinal digestion of yellow pea.

#### Seed microstructure

**Figure 3:**
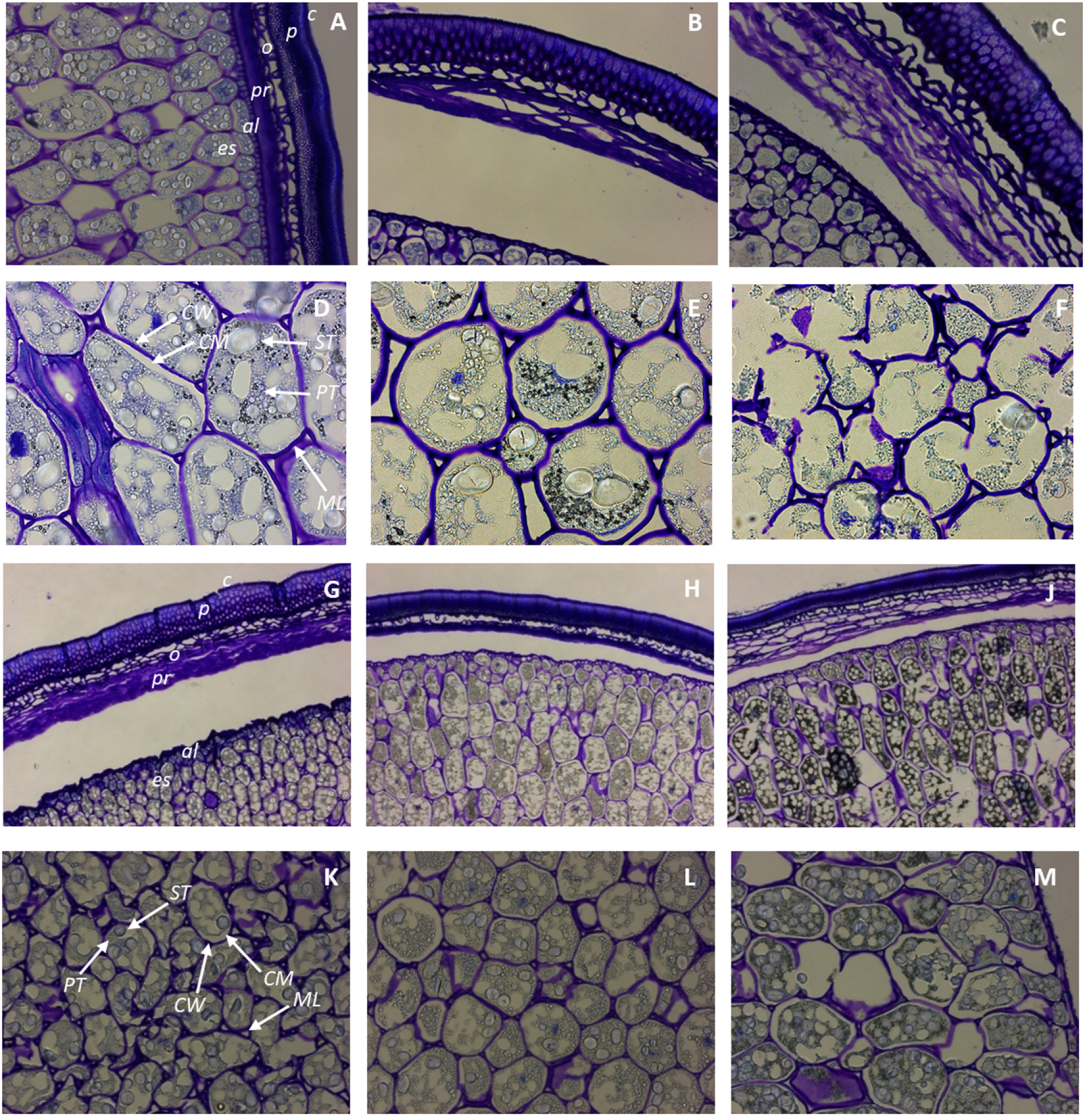
Panel A–F: Yellow pea visualised by Toluidine blue O. Top: seed coat (testa), brightfield, 20×. Bottom: cotyledon, brightfield, 40×. Panels A,D: soaked; B,E: germinated; Panels C,F: solid state fermented with L. plantarum. c: cuticle; p: palisade cells; o- osteosclereids; pr: parenchyma; al: aleurone; es: endosperm; CW: cell wall; CM: cell membrane; ML: middle lamella; ST: starch; PT: protein. Panels G–M: green lentils visualised by Toluidine blue O. Top: seed coat (testa), brightfield, 10×. Bottom: cotyledon, brightfield, 20×. Panels G,K: soaked; Panels H,L: germinated; Panels J,M: solid state fermented with L. plantarum.

**Figure 4:**
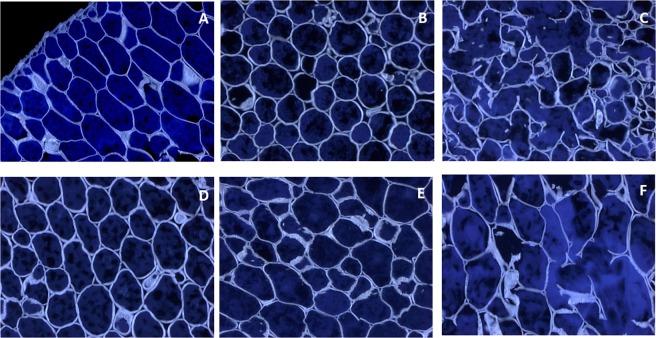
Yellow pea (Panels A–C) and green lentil (Panels D–F) visualised by Calcoflour White Stain, UV light, 20×. (Panels A,D): soaked; (Panels B,E): germinated; (Panels C,F): solid state fermented with L. plantarum.

The microstructures of the untreated, germinated and SSF yellow pea and green lentil are shown in Figs 3 and 4. In yellow peas, the cuticle (*c*), *linea lucida*, palisade layer (*p*) of osteosclereids (*o*) and macrosclereids, parenchyma (*pr*) and aleurone layer (*al*) could be identified (Fig. 3, Panel A). Germination and solid-state fermentation with *L. plantarum* had an evident effect on the seed coat composition. In both cases, macrosclereids and osteosclereids greatly increased in size, while parenchyma cells disappeared. Interestingly, the palisade cells turned into a lighter pinkish-purple colour as a result of SSF (Fig. 3, Panel C, J), possibly indicating the presence of carboxylated polysaccharides. Moreover, the *linea lucida* was no more evident in solid-state fermented yellow pea seed, which was expected after water imbibition. When considering the cotyledon of yellow pea seeds (Fig. 3, Panel D, E, F), cell wall (*CW*), cell membrane (*CM*), middle lamella (*ML*), starch (*ST*), and proteins (*PT*) could clearly be identified in the soaked seed (Fig. 3, Panel D). Germination caused an increase in cell wall thickness, depletion in starch and protein granules and increase in other protein-sized molecules which greatly stained with toluidine blue, possibly phenolic compounds. On the other hand, SSF with *L. plantarum* caused a more intense depletion of starch and storage proteins, and a breakage of the cell wall in certain regions of the seeds (Fig. 4). The same cellular structures were identified in green lentils (Fig. 3, Panel G-M). SSF with *L. plantarum* caused a similar effect on osteoscleroids, while macrosclereids appeared to be decreased in size in this case. In the cotyledon, bacterial fermentation only depleted starch granules and proteins in certain cells, but not in others. The cell membrane increased in size, and the cytosol turned into a greenish colour, indicating a change in compositional factors. Germination did not follow the same trend observed for yellow pea, and both seed coat and cell wall appeared thinned after germination (Fig. 3, Panel H, L).

## Discussion

Digestibility and nutritional value of legume seeds are primarily limited by the presence of anti-nutritional factors and the thick cell wall and seed coat, which represent physical barriers that limit the activity of digestive enzymes^39^. Food bioprocessing can influence the integrity of plant cell walls, dissociate the nutrient-matrix complexes ultimately improving nutrients bioavailability. Fermentation, heat treatment, germination and enzymatic hydrolysis prior to simulated digestion have been related to an increase in the release of antioxidants, DPP-IV inhibitors and improved protein digestibility in pulses^13–15,21–23^. In this paper, five bioprocessed pulse samples were subjected to simulated gastrointestinal digestion, and subsequently tested for their ability to inhibit DPP-IV and α-glucosidase, two key enzymes involved in human blood glucose regulation. The enzymatic assays were performed in simulated intestinal fluid (SIF)^40^ to simulate the human body environment. Yellow peas and green lentils were chosen as representative pulses for further investigation. Among the fermented samples, grinding combined with SSF was chosen as the preferred method, based on the bioactivity. The observed effects of germination and SSF on bioaccessibility of the bioactive compounds in common pulses are discussed separately.

### Germination

Germination triggers the displacement of storage proteins (legumins and vicilins), *de novo* synthesis of proteins, energy generation, synthesis of primary (amino acids, lipids, sugars) and secondary metabolites, signal molecules, rearrangement of the cellular architecture, and activation of defensive mechanisms to protect the seed from pathogens and oxidative damage caused by reactive oxygen species^28^. Interestingly, the storage protein convicilin that disappeared in the SDS-PAGE (Fig. 2, Panel B), plays a major role in the loss of desiccation tolerance, which initiates germination^28^. Bands that intensified after germination had molecular weight ranges of 27–39 kDa and 50–90 kDa, corresponding to proteins previously reported to increase during pea seed germination^28^. Green lentil protein profile was also characterized by a variety of polypeptide subunits in the molecular weight range of 17–150 kDa, with major subunits at 17–22 KDa (albumin and γ-vicilin fractions), 24–35 kDa (basic and acidic subunits of 11 S globulin), at 48 kDa (vicilin) and 70 kDa (convicilin)^37,41^ (Fig. 2, Panel C,D). Moreover, many studies reported that germination significantly increased phenolic compounds in a broad variety of edible seeds. The increase was not always linear, as different classes of phenolic compounds are synthesised in different quantities at different germination stages, according to their physiological functions^29,30^. *De novo* synthesis of flavonoids and phenolic acids can occur during germination, due to the phenylalanine-lyase (PAL) enzyme^42^, and possibly occurred in our study for flavonols (kaempferol derivatives), flavanones (naringenin), flavone (luteolin glycosides), hydroxybenzoic acids (syringic and vanillic acid) and hydroxycinnamic acids in yellow pea and green lentil. In fact, no major decreases was observed in phenolic compounds present at the initial stage of the germination (day 0). Similar to our study, an increase in total phenolic and flavonoids content was observed during germination of sweet corn, with a decrease in bound flavonoid content. The authors further analysed the phenolic profile and observed an increase in syringic acid, hydroxycinnamic acid and ferulic acid, but did not observe any change in phenolic composition^43^. In contrast, we observed a change in the phenolic composition during germination, and this was particularly evident in the yellow pea extract.

When considering the difference observed in the protein profiles (Fig. 2), phenolic profiles (Table 2) and seed microstructures (Figs 3 and 4) of yellow pea and green lentils, it is not surprising that the bioactivity measured (DPP-IV and α-glucosidase inhibition) substantially changed from one pulse to another (Fig. 1, Panels A, C). In fact, bioactivity is strictly related to the bioactive compound profiles, especially for phenolics and peptides. The interaction between enzyme and bioactive molecules, in this case phenolics or peptides, is highly specific and takes place only if specific structural requirements are present. For instance, peptides derived from germinated soybean proteins showed an increased DPP-IV and α-glucosidase inhibitory activities, while peptides generated from proteins of germinated cowpea bean were significantly less bioactive, when compared to their non-germinated forms^13,31^. The phenolic compounds identified in the two legume seeds were greatly different (Table 2), although belonging to the same classes (flavonoids, phenolic acids). This could explain why germination caused a decrease in the bioactivity of green lentils, but not yellow peas (Fig. 1, Panels A, C).

**Table 2:** Influence of bioprocessing on the content (μg/g DW) of phenolic compounds in green lentil and yellow pea extracts.

R_t_	Compound	Germination				Fermentation		
*Green Lentils*		Day 0	Day 3	Day 5	Day5+ *SGID*	Flour	F + SSF	F + SSF + *SGID*
10.211	2,5-Dihydroxybenzoic acid^a^	60.51	54.21	49.07	3.66	66.93	57.08	49.81
11.513	Catechin glucoside^b^	24.23	9.01	7.57	174.54	77.72	63.15	59.30
13.336	Catechin gallate^b^	28.91	27.71	26.98	20.89	34.04	31.94	20.59
16.280	Unknown^b^	ND	ND	8.11	8.06	6.85	5.74	5.05
17.368	Syringic acid^a^	11.35	8.41	8.55	12.39	24.73	7.89	9.70
23.122	*p*-couamric acid^a^	6.58	6.12	6.14	6.74	8.69	88.87	26.62
39.929	Kaempferol tetraglycoside^b^	139.07	112.86	93.94	70.89	125.85	93.35	66.13
40.628	Kaempferol triglycoside^b^	87.28	80.93	76.58	58.89	82.23	64.89	48.87
42.093	Kaempferol glucoside/Luteolin glucoside	ND	ND	7.98	7.04	7.84	42.95	31.58
45.158	Flavonoid derivative/Apigenin methyl ether^b^	5.38	28.14	38.85	4.29	7.58	5.95	ND
45.748	Flavonoid derivative^b^	14.35	22.25	31.36	25.73	16.21	11.45	9.06
46.489	Flavonoid derivative^b^	13.23	41.76	61.34	46.25	16.79	10.86	8.22
***Yellow Peas***		**Day 0**	**Day 3**	**Day 5**	**Day 5 + SGID**	**Flour**	**F + SSF**	**F + SSF + SGID**
2.711	Protocatechuic acid^b^	18.97	217.40	436.09	505.71	31.69	93.38	426.12
4.803	Dihydroxybenzoic acid^b^	7.98	19.55	27.89	30.63	12.57	22.31	16.01
13.021	Hydroxybenzoic acid^b^	17.63	6.30	6.00	5.35	11.72	10.82	9.81
15.493	Vanillic acid^b^	16.90	13.45	12.88	7.59	55.72	48.71	44.93
16.744	Chlorogenic acid^a^	ND	7.45	8.41	ND	ND	ND	ND
18.857	Luteolin glucoside^b^	20.03	15.65	13.90	4.62	78.82	55.00	ND
19.029	Luteolin glucoside^b^	N.D.	14.99	16.96	12.28	ND	ND	ND
28.17	Ferulic acid^a^	5.48	ND	ND	ND	5.90	ND	ND
42.451	Luteolin-6-C-glucoside/Kaempferol derivative^b^	22.19	31.50	51.96	15.23	26.03	19.37	18.08
47.19	Flavonoid derivative^b^	17.80	73.15	142.90	57.06	28.61	26.09	20.62
47.49	Naringenin^a^	49.28	50.61	54.97	50.54	49.22	53.16	49.24

^a^Compound quantified as μg/g DW of the corresponding standard.

^b^Compound quantified as Meqv rutin/g DW.

Germination was carried out for 0 (day 0), 3 (day 3), and 5 (day 5) days, and subsequently subjected to simulated gastrointestinal digestion (Day 5 + SGID). Fermentation was carried out for 0 (flour) and 48 h (F + SSF), and the fermented flour was subsequently subjected to simulated gastrointestinal digestion (F + SSF + SGID).

### Solid state fermentation

The metabolic activity of *L. plantarum* on cell wall components was evident in our study, with cell walls greatly disrupted at the end of the fermentation (Fig. 3, Panels C, J, and Fig. 4, Panels C,F), especially in yellow pea seed. SSF also affected the composition of the osteosclereids in the seed coat of both pulses, which resulted in a purple-violet colouration when stained with Toluidine blue. This specific coloration derives by binding of the dye to carboxylated compounds, and carboxylases have indeed been identified in *L. plantarum*^44^. In order to survive in its natural plant material habitat, *L. plantarum* developed a series of adaptive mechanisms, *i.e*. set of enzymes, that allow it to convert phenolics such as tannins into bioactive and less toxic compounds^34,45^. Bioconversion of glycosylated phenolic compounds to their aglycone forms was primarily related to the activity of bacterial β-glucosidase, which is a major enzyme involved in the breakage of the ester bonds between bound phenolic compounds and plant cell wall constituents, with the release of free and bound phenolics^46,47^. *L. plantarum* ATCC8014 indeed exhibited high β-glucosidase activity^48^. Deglycosylation and biotransformation by lactic acid bacteria was reported to significantly improve intestinal barrier integrity *in vitro*, and flavonoid bioavailability *in vivo*^49^. Similarly, in our study, kaempferol glycosides in green lentils were deglycosylated during SSF with *L. plantarum* (Table 2). The same glycoside activity was likely responsible for the significant increase in total phenolic content measured by colorimetric assay (Table 1).

The physical form of the substrate greatly affects the outcome of fermentation, making the nutrients required for bacterial growth more or less available. In our study, thermal treatment (121 °C, 15 min) of pulse seeds had a negative impact on bacterial growth. Intense heat treatment cause starch gelation, which impairs enzyme accessibility and could explain the low bacterial growth and low protein hydrolysis observed in all the heat-treated samples in our study. On the other hand, grinding seeds to flour is expected to increase the surface area for bacteria activity and therefore improves bacterial growth. The loss of viability observed during the fermentation of ground pulse samples could be related to the extensive fermentation from *L. plantarum*, providing a significantly higher ∆pH compared to the other pre-treatments, similar to previous findings^22,46,50,51^. When considering the protein profile, SSF led to a considerable decrease in band intensity possibly due to extensive proteolytic activity of *L. plantarum* on the pulse proteins and increase in protein fragments with a molecular weight below 10 kDa, or protein utilization by the bacteria. Proteolysis could be primarily induced by an acidic activation of endogenous proteinases, followed by the proteolytic activity of the *L. plantarum* to complete the hydrolysis leading to the formation of smaller peptides^52^.

In conclusion, germination and SSF with *L. plantarum* effectively altered the phenolic and protein profiles of yellow pea and green lentil, with improvement of their DPP-IV and α-glucosidase inhibitory activities. The microstructure of legume seeds was significantly modified by the bioprocesses. Particularly, SSF caused a significant degradation of the plant cell wall, which is a major barrier to nutrients digestibility for humans in plant derived foods. Further studies are needed to investigate the bioavailability of bioactive compounds in the bioprocessed pulses, and their ability to exert DPP-IV and α-glucosidase inhibitory activities *in vivo* after oral consumption.

## Material and Methods

### Materials

Chickpeas (*Cicer arietinum* variety Kabuli), Yellow peas (*Pisum sativum*), Faba beans (*Vicia faba*), Red beans (*Phaseolus vulgaris* variety Kidney), green lentils (*Lens culinaris*) were generously donated by Pulse Canada (Manitoba, Canada). α-Glucosidase from *Saccharomyces cerevisiae* (≥100 units/mg protein), dipeptidyl peptidase IV human (recombinant expressed in Sf9 cells), α-amylase (from porcine pancreas, type VI-B, ≥5 units/mg solid), pancreatin (from porcine pancreas, 8xUSP specification), pepsin (from porcine gastric mucosa, ≥250 units/mg solid), Calcoflour white stain (calcofluor white M2R 1 g/L, evans blue, 0.5 g/L), Toluidine blue O, gallic acid, vanillin and catechin were purchased from Millipore Sigma (Burlington, MA, USA). *Lactobacillus plantarum* ATCC® 8014™ was purchased from Cedarlane (Burlington, ON, Canada). DeMan, Rogosa and Sharpe (MRS) broth, M17 broth and Bacteriological agar were purchased from Oxoid (Nepean, ON, Canada). DC^TM^ Protein Assay Kit II and Ladder Precision Plus Protein dual color standards were purchased from BioRad (Mississauga, ON, Canada). GelCode blue safe protein stain was purchased from Fisher Scientific (Toronto, ON, Canada).

### Preparation of microbial culture

*Lactobacillus plantarum* (ATCC® 8014™) culture was reconstituted in MRS broth and stored at −80 °C in 30% (v/v) glycerol. Bacterial cells were propagated twice prior to experimental use, recovered by centrifugation (10000 *g* for 10 min at 4 °C) and washed twice in sterile saline solution (0.9% NaCl, w/v). The obtained suspension was used as inoculum for solid-state fermentation.

### Pulse bioprocessing

#### Solid state fermentation (SSF)

Pulse seeds were prepared in three different ways (i, ii, iii) prior to inoculation with *Lactobacillus plantarum* ATCC® 8014™. (i) Cracked beans (10 g) were sanitized for 10 min with 2% (v/v) sodium hypochlorite, rinsed 3 times with sterile distilled water and soaked overnight in sterile distilled water. On the following day, cracked seeds were homogeneously inoculated with 1 mL of *L. plantarum* suspension containing 9 log_10_ colony-forming units (CFU) mL^−1^. (ii) Cracked beans (10 g) were suspended in distilled water (1:2, w/v) and autoclaved at 121 °C for 15 min. After cooling down, the autoclaved beans were then homogeneously inoculated with 2 mL of *L. plantarum* suspension containing 9 log_10_ colony-forming units (CFU) mL^−1^. (iii) Pulses were ground into flour (10 g), mixed with equal volume (1:1, w/v) of sterile distilled water and homogeneously inoculated with 1 mL of *L. plantarum* suspension containing 9 log_10_ colony-forming units (CFU) mL^−1^. Fermentation of pulse products was carried out for 48 h at 37 °C. SSF samples were withdrawn at 0 and 48 h for microbiological analysis, then freeze-dried and stored at −20 °C for chemical analysis.

#### Germination

Pulses (20 g) were sanitized for 10 min with 2% (v/v) sodium hypochlorite and then rinsed 3 times with sterile distilled water and soaked overnight (15 hr) at room temperature (23 °C). On the following day, the excess water was removed and pulses were placed in a sterile petri dish covered with filter paper and sprayed with 5 ml distilled water. The germination was carried out at 0, 3 and 5 days at room temperature (21 °C) in the darkness. Seeds were then watered with 2 ml distilled sterile water on day 2 and 4 of germination. Germinated pulses were then frozen at −80 °C, freeze-dried, and ground to a flour for further analysis.

### Microbiological analysis

Fermentation was monitored by withdrawing samples at 0 and 48 h of fermentation using plate count and change in pH to determine the efficiency of the process. *L. plantarum* was counted in acidified MRS broth (pH 5.8) supplemented with 1.5% (w/v) agar, after anaerobic incubation at 30 °C for 72 h. Total anaerobic population was counted on reinforced clostridial agar (RCA) after anaerobic incubation at 30 °C for 48 h. Total aerobic population was counted on plate count agar (PCA) after incubation at 30 °C for 48 h. Cell counts were expressed as log_10_ CFU/ml. pH was measured after 0 and 48 h of fermentation by means of a pH meter (Fisherbrand, USA).

### Simulated gastrointestinal digestion

*In vitro* simulated gastrointestinal digestion (SGD) was conducted according to the consensus three steps *in vitro* digestion model described by Minekus *et al*.^40^. The entire process was performed at 37 °C. In brief, 5 g of ground sample were mixed with 5 mL of solution containing simulated salivary fluids (SSF) and salivary α-amylase (1500 U/ml) and incubated two minutes, with a pH of 7.0. The 10 mL of bolus were then mixed with 10 mL of a solution containing simulated gastric fluids (SGF) and porcine pepsin (25000 U/mL) and incubated for two hours with constant shaking (120 rpm), at a constant pH of 3.0. The gastric chyme was then mixed with an equal amount of a solution made of simulated intestinal fluids (SIF), pancreatin (100 trypsin U/mL), bile extract (10 mM) and incubated for two hours at constant pH of 7.0 and agitation (120 rpm). The amount of pepsin and pancreatin were determined based on their activities, as suggested by the consensus method^53^. Pepsin activity was assessed according to the method described by Anson *et al*.^54,55^. Trypsin activity was determined according to the method described by Hummel *et al*.^56^.

### Chemical analysis

#### Determination of total phenolic content (TPC)

Total phenolics were extracted following a modified method from Zhang *et al*.^19^. Briefly, 200 mg pulse flour were extracted in the dark with 70% MeOH containing 1% HCl (v/v) using a shaking incubator (Mandel, Canada) at 200 rpm for 3 h at 37 °C. The mixture was then centrifuged 7000 *g* for 15 min and supernatant was stored at −20 °C in the darkness for TPC analysis. Total phenolic content (TPC) in crude extract was determined by a colorimetric reaction using Folin and Ciocalteu’s phenol reagent according to the method described by Singleton *et al*.^57^, with modifications. Briefly, 50 µL gallic acid standard or pulse extract were mixed with 475 µL of 10-fold diluted Folin-Ciocalteu reagent in an amber microcentrifuge tube and reacted for 5 min at room temperature. A 475 µL of 60 g/L sodium carbonate (Na_2_CO_3_) solution was then added and incubated 2 h at room temperature before the absorbance was read at 725 nm using a visible-UV microplate reader (Tecan, Switzerland). Calibration was achieved with an aqueous gallic acid solution (31.25–500 µg/mL). The total phenolic content (TPC) was expressed as mg gallic acid equivalents (GAE) per gram dry weight pulse flour (mg GAE/g DW) based on the calibration curve.

#### Identification of phenolic compounds by HPLC-DAD

Chromatographic analysis were performed using a Breeze™ 2 HPLC system equipped with a 2998 photodiode detector and Empower™ 3 data analysis software from Waters Canada (Montreal, QC). The separation was performed on a Waters Spherisorb 5 µm ODS2 4.6 × 150 mm analytical column. The mobile phase consisted of 1% formic acid in water (v/v) (solvent A) and 95% methanol/5% acetonitrile (v/v) (solvent B). Injection volume 20 µL and flow rate was kept at 0.8 mL/min for a total run time of 60 min. A linear gradient solvent (A:B) system was used as follow: 0–5 min (100:0); 5–10 min (90:10); 10–25 min (80:20); 25–35 min (75:25); 35–45 min (70:30); 45–50 min (20–80); 50–60 min, (100:0). Data were collected at 280 and 320 nm. Phenolic compounds were identified by comparing retention time and UV absorption spectra with available commercial standards. Compounds with no standard reference material were tentatively identified by UV spectrum, retention time and by matching with published data.

#### Soluble protein content and SDS-PAGE profile

Soluble protein concentration of the samples was determined using DC™ Protein Assay (Bio-rad, California, USA). In brief, 100 mg of ground sample was weighted, mixed with 1 mL distilled water, and spinned for 10 min at 10 000 *g*. Five microliters of appropriately diluted supernatant were then placed in a 96 well plate, mixed with reagent A (alkaline copper tartrate solution) and 200 µL of reagent B (diluted Folin reagent). The plate was shacked for 5 s and allowed to stand for 15 min at room temperature, before reading the absorbance at 750 nm with a microplate reader (Tecan, Switzerland). A standard curve was prepared with bovine serum albumin (BSA) using concentrations of 0.08–1.23 mg/mL in deionised water and measured along with samples. The protein concentration was calculated using a BSA curve (y = 0.2777 × −0.0115, R^2^ = 0.9874).

Proteins from SSF and germination were separated by SDS-PAGE under reducing condition. In brief, protein extracts were prepared with sample buffer to a final concentration of 0.5 mg/mL of proteins, then denatured for 10 min at 95 °C and loaded onto a denaturing gel (6% to 18% acrylamide gradient), prior to electrophoresis (75 min, 120 V). The gel was then stained with GelCode^TM^ Blue Safe Protein Stain according to the manufacturer manual and visualised with ChemiDoc MP Imaging System (Bio-Rad, USA).

Bands intensity was quantified using the ImageJ software version 1.x (NIH, USA).

#### Determination of protein degree of hydrolysis (DH)

Degree of hydrolysis of proteins was determined according to the method described by Nielson *et al*.^58^, with some modifications. In brief, 20 mg of ground sample was weighted, mixed with 1 mL of distilled water, and spinned for 10 min at 10 000 *g*. In a 96-well plate, 30 µL of sample (supernatant), standard (serine), or control (deionized water) was mixed with 225 µL of OPA reagent. The mixture was incubated for 2 min at room temperature (23 °C), before reading the absorbance at 340 nm. Degree of hydrolysis was determined with Eq. (1) and expressed as meqv Ser-NH_2_/g DW.

1 $${\rm{S}}{\rm{e}}{\rm{r}}{\rm{i}}{\rm{n}}{\rm{e}}-{{\rm{N}}{\rm{H}}}_{2}=\frac{(ODsample-ODblank)}{(ODstandard-ODblank)}\times 0.9516\frac{meqv}{L}\times 0.1\times \frac{100}{x}$$

where serine-NH2 = meqv serine NH_2_ g sample; X = g sample; 0.1 is the sample volume in litre.

### Determination of enzyme inhibition activity

#### α-Glucosidase inhibition

α-Glucosidase inhibition was determined according to the method described by Mojica L. (2016)^59^, with modifications. In brief, 100 mg of sample was mixed with 1 mL of simulated intestinal fluid (SIF)^40^, shaking incubated for 10 min at 37 °C and 90 rpm, and centrifuged for 10 min at 10 000 *g*. The extract was then diluted in phosphate buffer (pH 6.9) to the final assay concentrations of 25, 12.5 and 6.25 mg/200 µL. Fifty microliters of the extract, control (phosphate buffer) or standard (1 mM Acarbose) were then transferred in triplicate in a 96-well microplate. 100 µL of 1 U/mL α-glucosidase solution and the mixture was incubated for 10 min at room temperature (23 °C). Fifty microliters of the substrate solution (5 mM p-nitrophenyl- α-D-glucopyranoside dissolved in 0.1 M sodium phosphate buffer, pH 6.9) was then added to each well, and the mixture was incubated for 5 min at 25 °C, before reading the absorbance at 405 nm in a microplate reader (Tecan, Switzerland). The percentage of α-Glucosidase inhibition was determined with Eq. (2):

2 $$\frac{Abs\,test\,(Abs\,test-Abs\,blank)-Abs\,control\,(Abs\,control-Abs\,blank)}{Abs\,control\,(Abs\,control-Abs\,blank)}\times 100$$

Values for α-glucosidase inhibition were then converted in milliequiv of Acarbose (Fig. 1).

#### DPP-IV Inhibition

DPP-IV inhibition was determined according to the method described by Lacroix *et al*.^60^ and Nongonierma & FitzGerald^61^, with modifications. In brief, 100 mg of sample was mixed with 1 mL of simulated intestinal fluid (SIF)^40^, shaking incubated for 10 min at 37 °C and 90 rpm, and spinned for 10 min at 10 000 *g*. The extract was then diluted in Tris-HCl buffer (pH8.0). Twenty-five microliters of the extract, control or standard (diprotin A) were then transferred in a 96-well microplate followed by addition of 25 µL of substrate (Gly-Pro-p-nitroanilide 1.6 mM) and incubation for 10 min at 37 °C. Fifty microliters of DPP-IV (1852 U/mL) were then added and the mixture was incubated for 60 min at 37 °C. At the end of the incubation, the absorbance was immediately read at 405 nm with a microplate reader (Tecan, Switzerland). DPP-IV inhibition in percentage was determined as: 100 × {1- [(A_405_ (test sample) − A_405_ (test sample blank)]/[(A_405_ (positive control) − A_405_ (negative control)]}, and expressed as milliequivalents of Diprotin A. Positive control was prepared with 25 µL SIF, 25 µL substrate, 50 µL DPP-IV. Negative control was prepared with 75 µL SIF and 25 µL substrate. A standard curve was prepared with increasing concentration (0.5–40 µM) of Diprotin A. Values for DPP-IV inhibition were then converted in millieqv of diprotin A (Fig. 1).

### Brightfield and fluorescence microscopy

The microstructure of bioprocessed samples was analysed under bright field and fluorescence microscopy. Samples were fixed for 90 min at 37 °C in FAA solution (2:1:17 of 10% formalin, glacial acetic acid, 75% ethanol), as described by Schichnes *et al*.^62^. Fixed samples were then dehydrated through a series of ethanol solutions, positioned in molds, embedded in paraffin and mounted on blocks for sectioning. Sections were cut to a thickness of 0.5 *µ*m with a vibratome and each section was positioned and dried on a slide. Before staining, paraffin was removed by ethylene and ethanol cycles, then washed in distilled water and air dried. For Toluidine Blue O staining, a 0.1% aqueous solution was prepared and sections were stained for 15 min, washed with water, and mounted on a glass slide with 25% glycerol solution. Samples were observed with brightfield microscopy (Carl Zeiss, Germany). For Calcoflour staining, a mixture of Calcoflour White Stain (Sigma-Aldrich, St. Louis, USA) and 10% potassium hydroxide (1:1) was placed on the specimen for one minute, before observation under UV light using DAPI channel (Carl Zeiss, Germany). Toluidine blue O is a polychromatic dye that reacts with various constituents of the plant cells, with each reaction leading to a different colour. A pinkish-purple colour is generated when Toluidine blue O reacts with carboxylated polysaccharides; bright blue when reacting with poly-aromatic compounds (e.g., lignin, tannins), and purplish-greenish blue with nucleic acids^63^. Calcoflour white is a fluorescent stain that strongly binds structures containing cellulose, callose, lignin, and other non- or weakly substituted β-glucans^63^.

### Statistical analysis

Experiments were carried out on three biological replicates, and each assay was further carried out in triplicate. Data were expressed as mean ± standard deviation (SD). Two-way analysis of variance (ANOVA) was used to compare different treatments on the same pulse. Tukey’s multiple comparison test was carried out to determine any significant differences between different sample treatments. Differences were considered significant when *p* < 0.05. Statistical analyses were carried out using GraphPad Prism 8.0 (GraphPad Software, La Jolla, CA, USA).

## Supplementary information

Supplementary information

## Data Availability

All data generated or analysed during this study are included in this article and its Supplementary Information Files.

## References

[1] American Diabetes Association, A. D. (2016). 3. Foundations of Care and Comprehensive Medical Evaluation. Diabetes Care.

[2] *Global report on diabetes. ISBN 978 92 4 156525 7* **978**, (WHO Library Cataloguing-in-Publication Data, 2016).

[3] Evans JM, Newton RW, Ruta DA, MacDonald TM, Morris AD (2000). Socio-economic status, obesity and prevalence of Type 1 and Type 2 diabetes mellitus. Diabet. Med..

[4] Chiasson J-L, Rabasa-Lhoret R (2004). Prevention of type 2 diabetes: insulin resistance and beta-cell function. Diabetes.

[5] Lacroix IME, Li-Chan ECY (2014). Overview of food products and dietary constituents with antidiabetic properties and their putative mechanisms of action: A natural approach to complement pharmacotherapy in the management of diabetes. Mol. Nutr. Food Res..

[6] Konrad B (2014). The Evaluation of Dipeptidyl Peptidase (DPP)-IV, α-Glucosidase and Angiotensin Converting Enzyme (ACE) Inhibitory Activities of Whey Proteins Hydrolyzed with Serine Protease Isolated from Asian Pumpkin (Cucurbita ficifolia). Int. J. Pept. Res. Ther..

[7] Wang Minghan (2011). Metabolic Syndrome.

[8] McIntosh, C. H. S., Kim, S.-J., Pederson, R. A., Heiser, U. & Demuth, H.-U. *Metabolic Syndrome (Underlying Mechanisms and Drug Therapies) || Dipeptidyl Peptidase IV Inhibitors for Treatment of Diabetes*. (John Wiley & Sons, Inc., Hoboken, New Jersey, 2011).

[9] Lacroix IME, Li-Chan ECY (2016). Food-derived dipeptidyl-peptidase IV inhibitors as a potential approach for glycemic regulation – Current knowledge and future research considerations. Trends Food Sci. Technol..

[10] Duez LNH, Cariou B, Staels B (2011). DPP-4 inhibitors in the treatment of type 2 diabetes. Biochem. Pharmacol..

[11] Stefano ED, Oliviero T, Udenigwe CC (2018). Functional significance and structure – activity relationship of food-derived a -glucosidase inhibitors. Curr. Opin. Food Sci..

[12] Mojica L, Meyer A, Berhow MA, de Mejía EG (2015). Bean cultivars (Phaseolus vulgaris L.) have similar high antioxidant capacity, *in vitro* inhibition of α-amylase and α-glucosidase while diverse phenolic composition and concentration. Food Res. Int..

[13] de Souza Rocha T, Hernandez LMR, Chang YK, de Mejía EG (2014). Impact of germination and enzymatic hydrolysis of cowpea bean (Vigna unguiculata) on the generation of peptides capable of inhibiting dipeptidyl peptidase IV. Food Res. Int..

[14] Mojica L, Chen K, de Mejía EG (2015). Impact of Commercial Precooking of Common Bean (Phaseolus vulgaris) on the Generation of Peptides, After Pepsin-Pancreatin Hydrolysis, Capable to Inhibit Dipeptidyl Peptidase-IV. J. Food Sci..

[15] Nongonierma A, FitzGerald R (2015). Investigation of the Potential of Hemp, Pea, Rice and Soy Protein Hydrolysates as a Source of Dipeptidyl Peptidase IV (DPP-IV) Inhibitory Peptides. Food Dig..

[16] Kim Bo-Ram, Kim Hyo, Choi Inhee, Kim Jin-Baek, Jin Chang, Han Ah-Reum (2018). DPP-IV Inhibitory Potentials of Flavonol Glycosides Isolated from the Seeds of Lens culinaris: In Vitro and Molecular Docking Analyses. Molecules.

[17] Xiao JB, Hogger P (2014). Dietary Polyphenols and Type 2 Diabetes: Current Insights and Future Perspectives. Curr. Med. Chem..

[18] Podsȩdek A, Majewska I, Kucharska AZ (2017). Inhibitory Potential of Red Cabbage against Digestive Enzymes Linked to Obesity and Type 2 Diabetes. J. Agric. Food Chem..

[19] Zhang B (2015). Phenolic profiles of 20 Canadian lentil cultivars and their contribution to antioxidant activity and inhibitory effects on α-glucosidase and pancreatic lipase. Food Chem..

[20] Higgins, J. A. Whole Grains, Legumes, and the Subsequent Meal Effect: Implications for Blood Glucose Control and the Role of Fermentation. *J. Nutr. Metab*. **2012** (2012). 10.1155/2012/829238 PMC3205742 22132324

[21] Zhang B (2014). Effect of domestic cooking on carotenoids, tocopherols, fatty acids, phenolics, and antioxidant activities of lentils (Lens culinaris). J. Agric. Food Chem..

[22] Limón RI (2015). Fermentation enhances the content of bioactive compounds in kidney bean extracts. Food Chem..

[23] Lee I-H, Hung Y-H, Chou C-C (2008). Solid-state fermentation with fungi to enhance the antioxidative activity, total phenolic and anthocyanin contents of black bean. Int. J. Food Microbiol..

[24] Runyon JR, Sunilkumar BA, Nilsson L, Rascon A, Bergenståhl B (2015). The effect of heat treatment on the soluble protein content of oats. J. Cereal Sci..

[25] Dhital S, Bhattarai RR, Gorham J, Gidley MJ (2016). Intactness of cell wall structure controls the *in vitro* digestion of starch in legumes. Food Funct..

[26] Capuano E (2017). The behavior of dietary fiber in the gastrointestinal tract determines its physiological effect. Crit. Rev. Food Sci. Nutr..

[27] Grundy MML (2016). Re-evaluation of the mechanisms of dietary fibre and implications for macronutrient bioaccessibility, digestion and postprandial metabolism. Br. J. Nutr..

[28] Wang WQ, Møller IM, Song SQ (2012). Proteomic analysis of embryonic axis of Pisum sativum seeds during germination and identification of proteins associated with loss of desiccation tolerance. J. Proteomics.

[29] Huang X, Cai W, Xu B (2014). Kinetic changes of nutrients and antioxidant capacities of germinated soybean (glycine max l.) and mung bean (vigna radiata l.) with germination time. Food Chem..

[30] Huang X (2014). Impact of germination on phenolic content and antioxidant activity of 13 edible seed species. Food Chem..

[31] González-Montoya, M., Hernández-Ledesma, B., Mora-Escobedo, R. & Martínez-Villaluenga, C. Bioactive Peptides from Germinated Soybean with Anti-Diabetic Potential by Inhibition of Dipeptidyl Peptidase-IV, α-Amylase, and α-Glucosidase Enzymes. *Int. J. Mol. Sci. Artic*. **19** (2018). 10.3390/ijms19102883 PMC6213256 30249015

[32] Rizzello CG (2015). Italian legumes: effect of sourdough fermentation on lunasin-like polypeptides. Microb. Cell Fact..

[33] Bartkiene E, Krungleviciute V, Juodeikiene G, Vidmantiene D, Maknickiene Z (2015). Solid state fermentation with lactic acid bacteria to improve the nutritional quality of lupin and soya bean. J. Sci. Food Agric..

[34] Felipe FLD, Rivas BD, Muñoz R (2017). Differential Gene Expression by Lactobacillus plantarum WCFS1 in Response to Phenolic Compounds Degradation Reveals New Genes Involved in Tannin Degradation. Appl. Environ. Microbiol..

[35] Ma Z, Boye JI, Hu X (2018). Nutritional quality and techno-functional changes in raw, germinated and fermented yellow field pea (Pisum sativum L.) upon pasteurization. LWT - Food Sci. Technol..

[36] Liang HN, Tang CH (2013). PH-dependent emulsifying properties of pea [Pisum sativum (L.)] proteins. Food Hydrocoll..

[37] Ladjal-Ettoumi Y, Boudries H, Chibane M, Romero A (2016). Pea, Chickpea and Lentil Protein Isolates: Physicochemical Characterization and Emulsifying Properties. Food Biophys..

[38] Tzitzikas EN, Vincken JP, De Groot J, Gruppen H, Visser RGF (2006). Genetic variation in pea seed globulin composition. J. Agric. Food Chem..

[39] Melito C, Tovar J (1995). Cell walls limit *in vitro* protein digestibility in processed legume seeds. Food Chem..

[40] Minekus M (2014). A standardised static *in vitro* digestion method suitable for food - an international consensus. Food Funct..

[41] Barbana C, Boye JI (2011). Angiotensin I-converting enzyme inhibitory properties of lentil protein hydrolysates: Determination of the kinetics of inhibition. Food Chem..

[42] Ren S-C, Sun J-T (2014). Changes in phenolic content, phenylalanine ammonia-lyase (PAL) activity, and antioxidant capacity of two buckwheat sprouts in relation to germination. J. Funct. Foods.

[43] Xiang Nan, Guo Xinbo, Liu Fengyuan, Li Quan, Hu Jianguang, Brennan Charles Stephen (2017). Effect of Light- and Dark-Germination on the Phenolic Biosynthesis, Phytochemical Profiles, and Antioxidant Activities in Sweet Corn (Zea mays L.) Sprouts. International Journal of Molecular Sciences.

[44] Kiatpapan P., Kobayashi H., Sakaguchi M., Ono H., Yamashita M., Kaneko Y., Murooka Y. (2001). Molecular Characterization of Lactobacillus plantarum Genes for  -Ketoacyl-Acyl Carrier Protein Synthase III (fabH) and Acetyl Coenzyme A Carboxylase (accBCDA), Which Are Essential for Fatty Acid Biosynthesis. Applied and Environmental Microbiology.

[45] Rodríguez H, Rivas B, Gómez-Cordovés C, Muñoz R (2008). Degradation of tannic acid by cell-free extracts of Lactobacillus plantarum. Food Chem..

[46] Torino MI (2013). Antioxidant and antihypertensive properties of liquid and solid state fermented lentils. Food Chem..

[47] Lee Y, Oh J, Jeong YS (2015). Lactobacillus plantarum-mediated conversion of flavonoid glycosides into flavonols, quercetin, and kaempferol in Cudrania tricuspidata leaves. Food Sci. Biotechnol..

[48] Landete JM, Curiel JA, Rodríguez H, de las Rivas B, Muñoz R (2014). Aryl glycosidases from Lactobacillus plantarum increase antioxidant activity of phenolic compounds. J. Funct. Foods.

[49] Shimojo Y, Ozawa Y, Toda T, Igami K, Shimizu T (2018). Probiotic Lactobacillus paracasei A221 improves the functionality and bioavailability of kaempferol-glucoside in kale by its glucosidase activity. Sci. Rep..

[50] Giraud E, Lelong B, Raimbault M (1991). Influence of pH and initial lactate concentration on the growth of Lactobacillus plantarum. Appl. Microbiol. Biotechnol..

[51] Pieterse B, Leer RJ, J Schuren FH, van der Werf MJ (2005). Unravelling the multiple effects of lactic acid stress on Lactobacillus plantarum by transcription profiling. Microbiology.

[52] Rizzello, C. G. *et al*. Italian legumes: Effect of sourdough fermentation on lunasin-like polypeptides. *Microb. Cell Fact*., 10.1186/s12934-015-0358-6 (2015). 10.1186/s12934-015-0358-6 PMC4618940 26494432

[53] Marie, A. Supplemenary material - all enzyme assays - to Minekus *et al*. 2014 consensus *in vitro* digestion 2014 food & function. (2016).

[54] Anson ML (1938). The estimation of pepsin, trypsin, papain, and cathepsin with hemoglobin. J. Gen. Physiol..

[55] Anson ML, Mirsky AE (1932). The estimation of Pepsin with hemoglobin. J. Gen. Physiol..

[56] Hummel Brian C. W. (1959). A MODIFIED SPECTROPHOTOMETRIC DETERMINATION OF CHYMOTRYPSIN, TRYPSIN, AND THROMBIN. Canadian Journal of Biochemistry and Physiology.

[57] Singleton VL, Rossi JA (1965). Colorimetry of total phenolics with phosphomolybdic-phosphotungstic acid reagents. Am. J. Enol. Vitic..

[58] Nielsen P, Petersen D, Dambmann C (2001). Improved Method for Determining Food Protein Degree of Hydrolysis. J. Food Sci..

[59] Mojica L, de Mejía EG (2016). Optimization of enzymatic production of anti-diabetic peptides from black bean (Phaseolus vulgaris L.) proteins, their characterization and biological potential. Food Funct..

[60] Lacroix IME, Li-Chan ECY (2013). Inhibition of dipeptidyl peptidase (DPP)-IV and α-glucosidase activities by pepsin-treated whey proteins. J. Agric. Food Chem..

[61] Nongonierma AB, FitzGerald RJ (2013). Dipeptidyl peptidase IV inhibitory and antioxidative properties of milk protein-derived dipeptides and hydrolysates. Peptides.

[62] Schichnes D, Nemson JA, Ruzin SE (2005). Microwave Protocols for Plant and Animal Paraffin Microtechnique. Time.

[63] Mitra P, Loqué P (2014). Histochemical Staining of Arabidopsis thaliana Secondary Cell Wall Elements. J. Vis. Exp.

