# Supplementary material for: Bioprocessing of common pulses changed seed microstructures, and improved dipeptidyl peptidase-IV and α-glucosidase inhibitory activities
Source: Sci Rep. 2019 Oct 25;9:15308. doi: 10.1038/s41598-019-51547-5 (PMC6814730; doi:10.1038/s41598-019-51547-5)

**Supplementary information**

**Bioprocessing of common pulses changed seed microstructures, and improved dipeptidyl peptidase-IV and α-glucosidase inhibitory activities**

Elisa Di Stefano^1,2^, Apollinaire Tsopmo^3^, Teresa Oliviero^2^, Vincenzo Fogliano^2^, Chibuike C. Udenigwe^1,4,*^

^1^ School of Nutrition Sciences, University of Ottawa, Ottawa, Ontario, K1N 5E3, Canada

^2^ Food Quality and Design Group, Wageningen University and Research, P.O. Box 8129, 6700 EV, Wageningen, The Netherlands

^3^ Food Science and Nutrition Program, Department of Chemistry, Carleton University, Ottawa, Ontario K1S 5B6, Canada

^4^ Department of Chemistry and Biomolecular Sciences, University of Ottawa, Ottawa, Ontario, K1N 5E3, Canada

*Corresponding author: [cudenigw@uottawa.ca](mailto:cudenigw@uottawa.ca)

Supplementary Table S1

Table S1. Effect of bioprocessing on DPP-IV inhibition in five pulses, at a final concentration of 25 mg/mL (mean ± SD, n=3). Germination was performed for 0 (Day 0), 3 (Day 3) and 5 (Day 5) days in the darkness at 23°C. Solid state fermentation (SSF) with L.plantarum for 48 hours at 37°C. Prior SSF, samples were either soaked (S) overnight, heat treated 121°C for 15 min (HT), or grinded into flour (G). DPP-IV inhibition was measured at the sample concentration of 25 mg/mL. All samples were digested in vitro. For each row and bioprocessing (germination and fermentation), values marked with the same letter are significantly different (p < 0.05).

|  | GERMINATION | | | SOLID STATE FERMENTATION | | | | | |
| --- | --- | --- | --- | --- | --- | --- | --- | --- | --- |
| Pulse | **Day 0** | **Day 3** | **Day 5** | **S** | **S +SSF** | **HT** | **HT +SSF** | **G** | **G +SSF** |
| Chickpea | 57.4 ± 3.1^a^ | 59.6 ± 3.3^ab^ | 57.5 ± 5.1^b^ | 57.2 ± 9.3 | 52.1 ± 2.0^a^ | 60.6 ± 1.2 | 65.7 ± 2.0^a^ | 56.5 ± 1.6 | 56.1 ± 2.9 |
| Faba Bean | 61.1 ± 9.2^a^ | 65.7 ± 2.6 | 68.2 ± 2.7^a^ | 60.8 ± 5.9 | 63.3 ± 3.5 | 67.4 ± 6.9^a^ | 62.6 ± 4.4 | 61.8 ± 4.8 | 56.1 ± 3.0^a^ |
| Kidney Bean | 58.5 ± 1.0 | 47.1 ± 0.1 | 56.4 ± 0.8 | 45.7 ± 7.1^ab^ | 42.3 ± 0.7^cd^ | 66.1 ± 5.4^ace^ | 63.7 ±0.5^bde^ | 50.0 ± 9.5^e^ | 34.0 ± 2.8^ae^ |
| Green Lentil | 69.7 ± 2.0^a^ | 64.0 ± 1.2 | 61.1 ± 1.8^a^ | 63.9 ± 1.2 | 66.0 ± 3.5 | 66.6 ± 1.3 | 64.4 ± 2.6 | 56.9 ± 6.0 | 65.1 ± 1.1 |
| Yellow Pea | 55.1 ± 1.5 | 51.9 ± 0.8 | 53.3 ± 2.4 | 55.3 ± 4.2 | 61.2 ± 2.4 | 60.8 ± 1.1 | 60.2 ± 4.2 | 54.0 ± 2.6 | 57.8 ± 2.0 |

Supplementary Table S2

Table S2. Effect of bioprocessing on α-glucosidase inhibition in five pulses, at a final concentration of 25 mg/mL (mean ± SD, n=3). Germination was performed for 0 (Day 0), 3 (Day 3) and 5 (Day 5) days in the darkness at 23°C. Solid state fermentation (SSF) with L.plantarum for 48 hours at 37°C. Prior SSF, samples were either soaked (S) overnight, heat treated 121°C for 15 min (HT), or grinded into flour (G). All samples were digested in vitro. α-Glucosidase inhibitory activity was monitored at three different sample concentrations (25, 12.5 and 6.25 mg/mL For each row and bioprocessing (germination and fermentation), values marked with the same letter are significantly different (p < 0.05).

|  |  | GERMINATION | | | SOLID STATE FERMENTATION | | | | | |
| --- | --- | --- | --- | --- | --- | --- | --- | --- | --- | --- |
| Pulse | **[mg/mL]** | **Day 0** | **Day 3** | **Day 5** | **S** | **S +SSF** | **HT** | **HT +SSF** | **G** | **G +SSF** |
| Chickpea | **25** | 39.0 ± 10.1^ab^ | 75.3 ± 5.7^a^ | 70.3 ± 4.7^b^ | 52.9 ± 5.3^a^ | 53.2 ± 4.0^b^ | 78.0 ± 3.7^abcd^ | 68.9 ± 8.9^abcd^ | 52.6 ± 6.0^c^ | 45.7 ± 5.6^d^ |
|  | **12.5** | 19.2 ± 7.3 | 55.0 ± 8.7 | 52.2 ± 4.1 | 28.1 ± 4.7 | 29.8 ± 5.6 | 41.4 ± 2.8 | 41.0 ± 2.7 | 31.3 ± 8.4 | 24.0 ± 2.4 |
|  | **6.25** | 12.8 ±5.0 | 40.8 ± 13.4 | 43.3 ± 5.2 | 13.2 ± 6.1 | 13.2 ± 5.8 | 17.6 ± 2.9 | 15.6 ± 5.5 | 23.2 ± 15.5 | 17.2 ± 2.8 |
| Faba Bean | **25** | 66.6 ± 2.8 | 62.5 ± 3.2 | 56.4 ± 5.2 | 55.1 ± 13.2^ab^ | 49.8 ± 3.5^c^ | 82.5 ± 14.8^acde^ | 68.3 ± 1.3^bc^ | 55.7 ± 1.4^d^ | 58.5 ± 2.4^e^ |
|  | **12.5** | 27.9 ± 0.6 | 30.5 ± 5.6 | 30.9 ± 7.8 | 32.0 ± 13.1 | 22.0 ± 3.6 | 41.8 ± 3.2 | 40.9 ± 4.1 | 34.2 ± 9.0 | 37.5 ± 7.5 |
|  | **6.25** | 10.2 ± 4.8 | 15.6 ± 2.8 | 19.2 ± 7.4 | 10.9 ± 13.9 | 4.7 ± 4.2 | 17.9 ± 3.4 | 20.0 ± 6.8 | 20.8 ± 17.3 | 23.8 ± 15.7 |
| Kidney Bean | **25** | 33.2 ± 4.8 | 31.4 ± 2.7 | 40.4 ± 15.8 | 39.4 ± 15.9^a^ | 51.2 ± 3.5^b^ | 84.6 ± 13.0^abcd^ | 63.6 ± 1.1 | 45.1 ± 23.1^c^ | 50.5 ± 16.0^d^ |
|  | **12.5** | 5.3 ± 4.6 | 7.7 ± 0.5 | 13.8 ± 17.0 | 28.9 ± 12.6 | 38.2 ± 1.8 | 65.7 ± 7.4 | 36.5 ± 2.7 | 35.8 ± 12.9 | 11.5 ± 10.7 |
|  | **6.25** | ND | 1.2 ± 1.7 | 8.9 ± 15.4 | 23.6 ± 8.7 | 27.5 ± 6.3 | 61.1 ± 30.6 | 45.5 ± 8.1 | 25.3 ± 11.7 | 1.8 ± 3.1 |
| Green Lentil | **25** | 73.3 ± 0.6^ab^ | 57.7 ± 7.9^a^ | 49.5 ± 2.4^b^ | 44.0 ± 8.7^ab^ | 42.6 ± 5.7^cd^ | 77.5 ± 2.8^ace^ | 86.6 ± 22.4^bdf^ | 47.6 ± 18.7^ef^ | 65.6 ± 2.4^e^ |
|  | **12.5** | 32.1 ± 4.6 | 24.7 ± 2.7 | 25.1 ±1.3 | 26.9 ± 3.1 | 18.7 ± 6.4 | 44.1 ± 1.4 | 45.7 ± 7.3 | 35.4 ± 3.6 | 35.5 ± 0.3 |
|  | **6.25** | 5.5 ± 8.6 | 11.6 ± 7.2 | 8.8 ± 9.1 | 15.9 ± 9.1 | 9.1 ± 9.6 | 30.0 ± 2.8 | 25.7 ± 6.6 | 23.4 ± 15.0 | 14.0 ± 5.1 |
| Yellow Pea | **25** | 56.5 ± 5.2 | 56.0 ± 1.9 | 61.6 ± 9.7 | 55.3 ±10.4^ab^ | 56.5 ± 3.8^cd^ | 88.2 ± 5.0^acef^ | 77.8 ± 13.4^bdgh^ | 41.0 ± 5.0^eg^ | 58.1 ± 9.1^fh^ |
|  | **12.5** | 20.6 ± 5.0 | 25.1 ± 6.2 | 35.5 ± 5.4 | 27.1 ± 2.6 | 23.4 ± 2.3 | 57.7 ± 2.3 | 55.2 ± 7.1 | 26.4 ± 6.0 | 36.1 ± 3.8 |
|  | **6.25** | 5.3 ± 6.8 | 12.7 ± 5.3 | 14.7 ± 0.9 | 17.2 ± 13.1 | ND | 20.9 ± 4.7 | 27.6 ± 6.8 | 15.0 ± 4.6 | 16.5 ± 5.1 |

Supplementary Table S3

Table S3. Phenolic compounds identified by HPLC-DAD in green lentil and yellow pea extracts, based on retention time (R_t_), UV spectra, λ_max_, or corresponding commercial standards.

| Peak no. | Time (min) | Phenolics | Abs of detection | ʎ_max_ (nm) | Identification |
| --- | --- | --- | --- | --- | --- |
| *Green Lentil* | | | | | |
| 1 | 10.211 | 2,5-Dihydroxybenzoic acid | 320 | 224, 326 | Rt, UV, standard |
| 2 | 11.513 | Catechin glucoside | 280 | 224, 278 | UV, ʎ_max_ (^17^,^21^) |
| 3 | 13.336 | Catechin gallate | 280 | 224, 277 | UV, ʎ_max_ (^17^) |
| 4 | 16.280 | Unknown compound | 320 | 224, 273 | Rt, UV |
| 5 | 17.368 | Syringic acid | 320 | 224, 272 | Rt, UV, standard |
| 6 | 23.122 | p-coumaric acid | 320 | 225, 271, 308 | Rt, UV, standard, ʎ_max_ (^21^) |
| 7 | 39.929 | Kaempferol tetraglycoside | 320 | 224, 265, 346 | UV, ʎ_max_ (^17^,^21^) |
| 8 | 40.628 | Kaempferol triglycoside | 320 | 224, 265, 346 | UV, ʎ_max_ (^17^,^21^) |
| 9 | 42. 093 | Kaempferol glucoside/ Luteolin glucoside | 320 | 225, 265, 348 | UV, ʎ_max_ (^17^,^21^) |
| 10 | 45.158 | Flavonoid derivative/ Apigenin methyl ether | 320 | 225, 269, 328 | UV, ʎ_max_ (^17^,^21^) |
| 11 | 45.748 | Flavonoid derivative | 320 | 225, 269, 318 | UV, ʎ_max_ (^17^) |
| 12 | 46.489 | Flavonoid derivative | 320 | 225, 269, 316 | UV, ʎ_max_ (^17^) |
| *Yellow Pea* | | | | | |
| 1 | 2.711 | Protocatechuic acid | 280 | 215, 264 | UV, ʎ_max_ (^22^, ^23^, ^24^) |
| 2 | 4.803 | Dihydroxybenzoic acid | 280 | 224, 259 | UV, ʎ_max_ (^17^) |
| 3 | 13.021 | Hydroxybenzoic acid | 320 | 224, 274 | UV, ʎ_max_ (^24^) |
| 4 | 15.493 | Vanillic acid | 320 | 224, 289 | UV, ʎ_max_ (^22, 25^) |
| 5 | 16.744 | Chlorogenic acid | 320 | 224, 328 | Rt, UV, standard |
| 6 | 18.857 | Luteolin glucoside | 320 | 228, 284, 320, 364, 396 | UV, ʎ_max_ (^24^, ^26^) |
| 7 | 19.029 | Luteolin glucoside | 320 | 224, 271, 320, 390 | UV, ʎ_max_ (^24^, ^26^) |
| 8 | 28.17 | Ferulic acid | 320 | 224, 274, 320 | Rt, UV, standard, ʎ_max_ (^22^, ^27^) |
| 9 | 42.451 | Luteolin-6-C-glucoside/ Kaempferol derivative | 320 | 225, 266, 346 | UV, ʎ_max_ (^24^, ^17^, ^28^) |
| 10 | 47.19 | Flavonoid derivative | 320 | 225, 273, 320 | UV, ʎ_max_ (^17^) |
| 11 | 47.49 | Naringenin | 320 |  | Rt, UV, standard |

Supplementary Figure S1


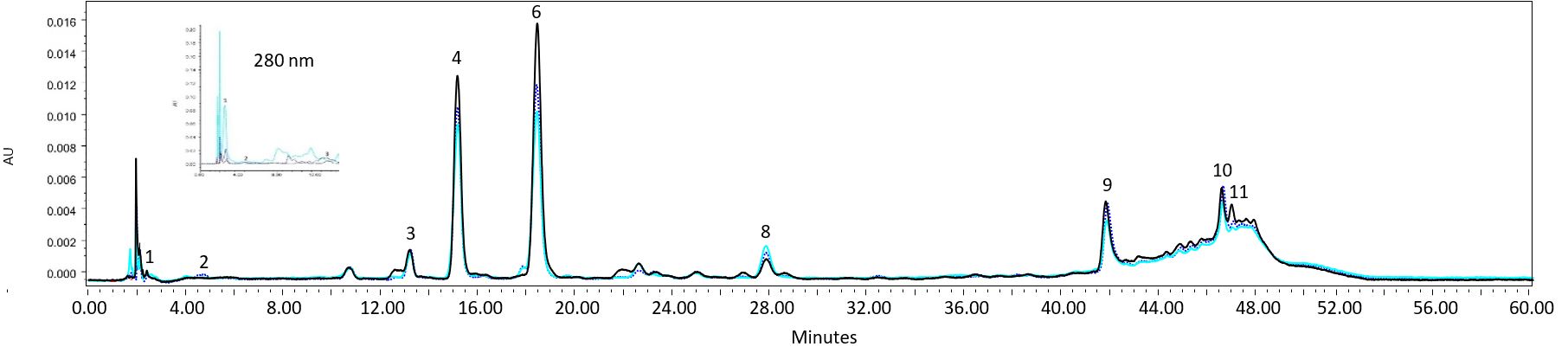


b


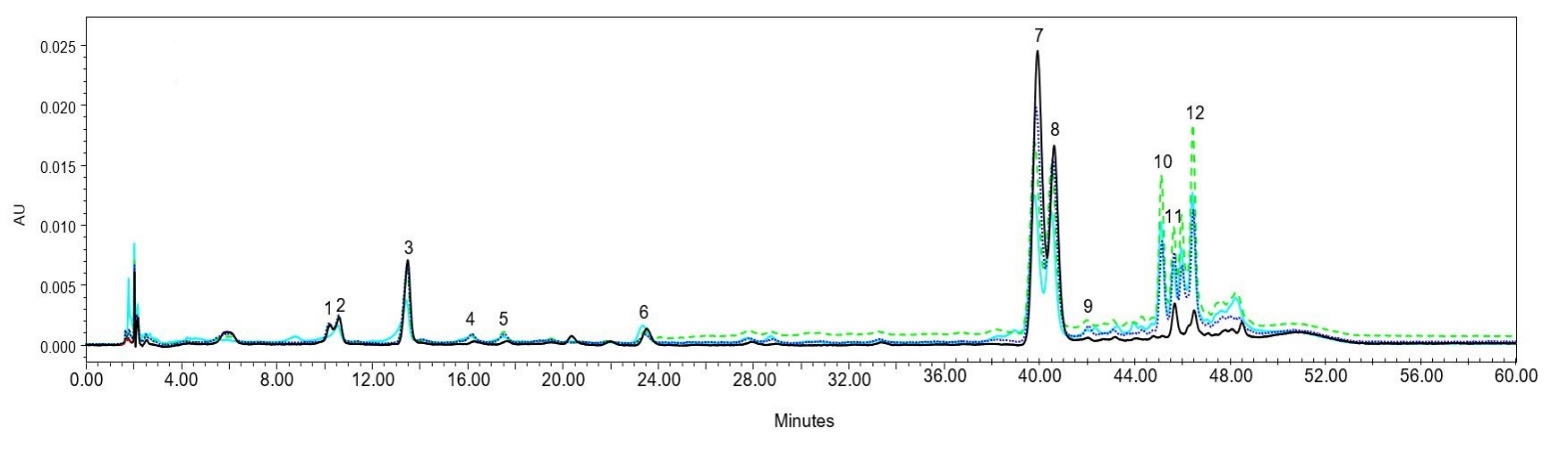


c


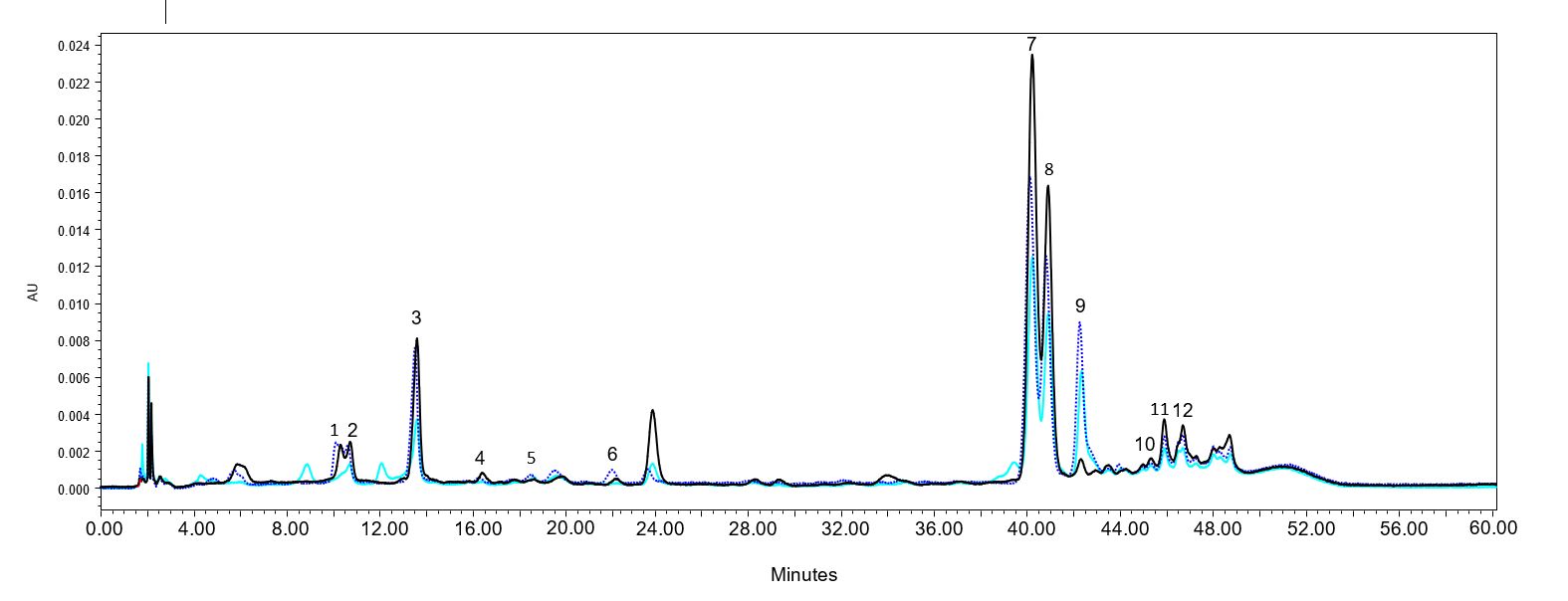


d


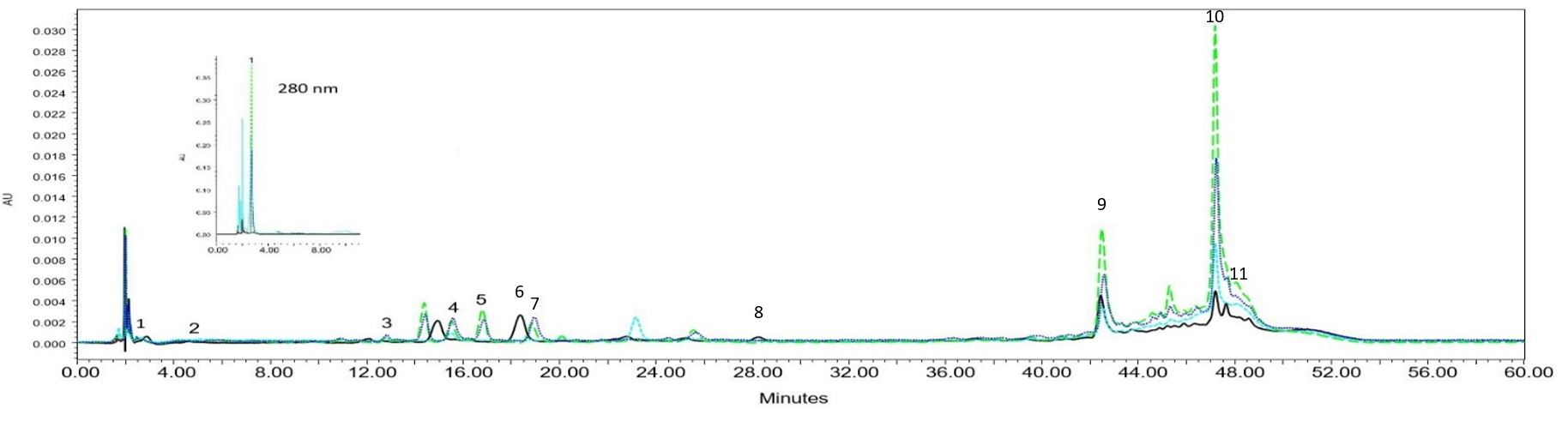


a

Figure S1. HPLC chromatograms of phenolic extracts of geminated (a,c) and fermented (b,d) yellow peas and green lentils, as detected at 320 nm, and with detail at 280 nm. Peaks without a number were not identified. Panel a & c, germination: black line: day 0, blue line: day 3, green line: day 5, light blue line: day 5 followed by simulated gastrointestinal digestion. Panel b & d, SSF with L. plantarum: black line: untreated flour, blue line: SSF flour, light blue line: SSF flour followed by simulated gastrointestinal digestion.

Original gels for Figure 2

Fig. 2, Gel A


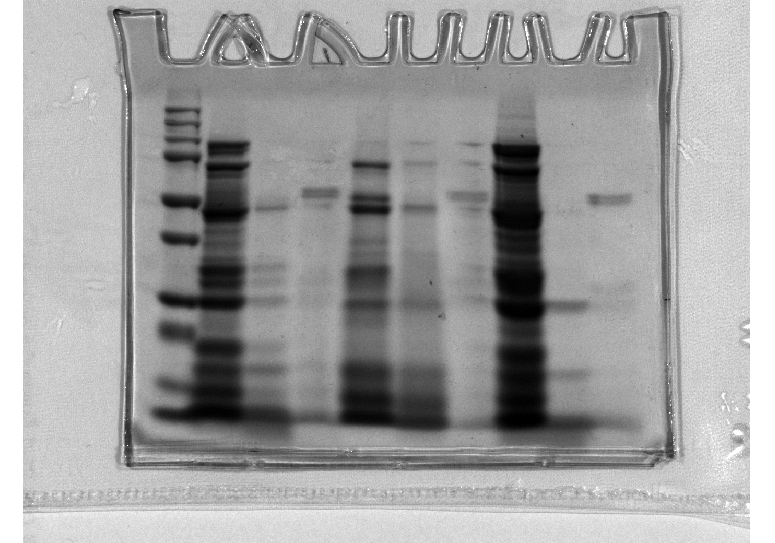


Fig. 2,
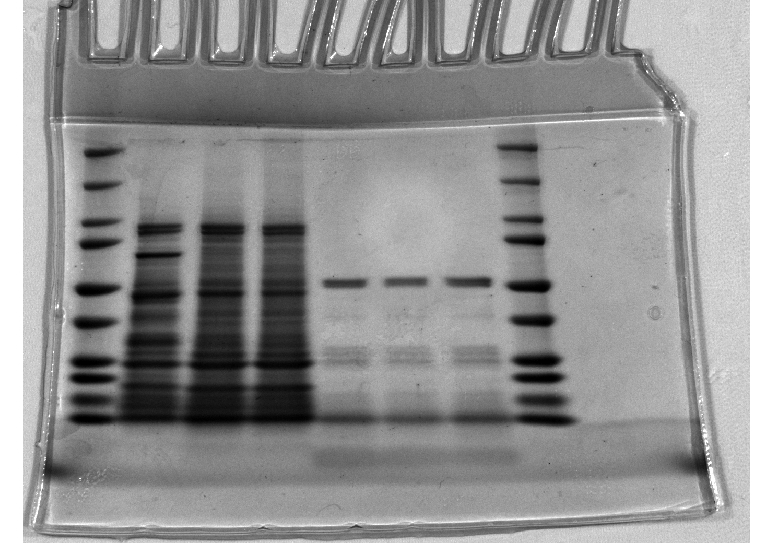
Gel B

Fig. 2,
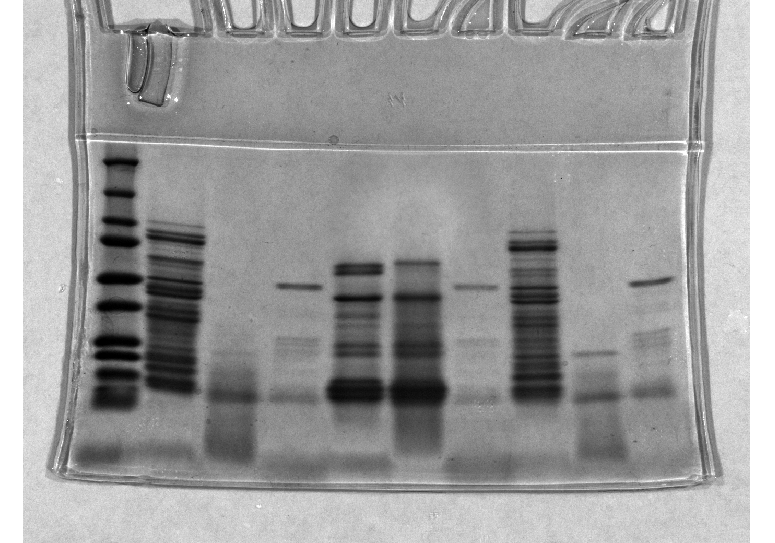
Gel C

Fig. 2, Gel D


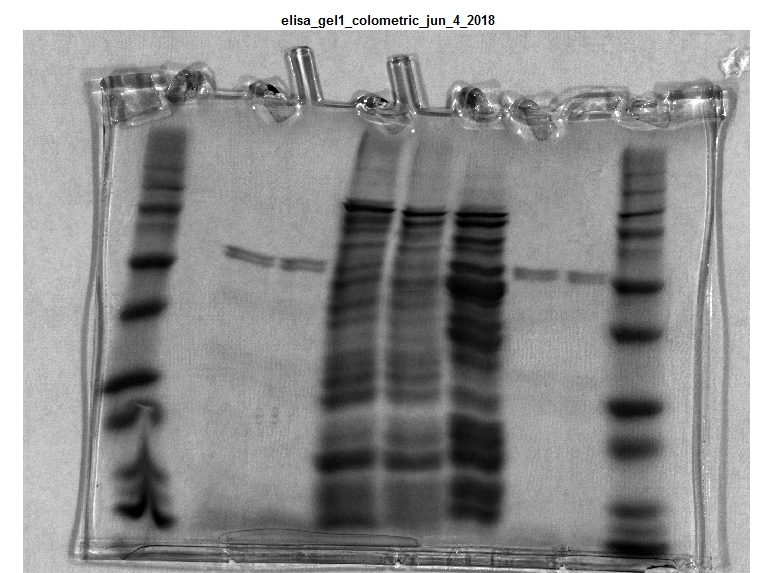

Supplement: Supplementary file 1 — Supplementary information [file 41598_2019_51547_MOESM1_ESM.docx]
